# Oct4 Is Required ∼E7.5 for Proliferation in the Primitive Streak

**DOI:** 10.1371/journal.pgen.1003957

**Published:** 2013-11-14

**Authors:** Brian DeVeale, Irina Brokhman, Paria Mohseni, Tomas Babak, Charles Yoon, Anthony Lin, Kento Onishi, Alexey Tomilin, Larysa Pevny, Peter W. Zandstra, Andras Nagy, Derek van der Kooy

**Affiliations:** 1 Department of Molecular Genetics, University of Toronto, Toronto, Ontario, Canada; 2 Department of Biology, Stanford University, Stanford, California, United States of America; 3 Institute of Biomaterials and Biomedical Engineering, University of Toronto, Toronto, Ontario, Canada; 4 Institute of Cytology, Russian Academy of Science, St-Petersburg, Russia; 5 Department of Genetics, University of North Carolina, Chapel Hill, North Carolina, United States of America; University of Wisconsin, United States of America

## Abstract

Oct4 is a widely recognized pluripotency factor as it maintains Embryonic Stem (ES) cells in a pluripotent state, and, *in vivo*, prevents the inner cell mass (ICM) in murine embryos from differentiating into trophectoderm. However, its function in somatic tissue after this developmental stage is not well characterized. Using a tamoxifen-inducible Cre recombinase and floxed alleles of Oct4, we investigated the effect of depleting Oct4 in mouse embryos between the pre-streak and headfold stages, ∼E6.0–E8.0, when Oct4 is found in dynamic patterns throughout the embryonic compartment of the mouse egg cylinder. We found that depletion of Oct4 ∼E7.5 resulted in a severe phenotype, comprised of craniorachischisis, random heart tube orientation, failed turning, defective somitogenesis and posterior truncation. Unlike in ES cells, depletion of the pluripotency factors Sox2 and Oct4 after E7.0 does not phenocopy, suggesting that ∼E7.5 Oct4 is required within a network that is altered relative to the pluripotency network. Oct4 is not required in extraembryonic tissue for these processes, but is required to maintain cell viability in the embryo and normal proliferation within the primitive streak. Impaired expansion of the primitive streak occurs coincident with Oct4 depletion ∼E7.5 and precedes deficient convergent extension which contributes to several aspects of the phenotype.

## Author Summary

Embryogenesis is an intricate process requiring that division, differentiation and position of cells are coordinated. During mammalian development early pluripotent populations are canalized or restricted in potency during embryogenesis. Due to considerable interest in how this fundamental state of pluripotency is maintained, and the requirement of the transcription factor Oct4 to maintain pluripotency, Oct4 has been intensively studied in culture. However, it is not clear what role Oct4 has during lineage specification of pluripotent cells. Oct4 removal during lineage specification indicates that it is required in the primitive streak of mouse embryos to maintain proliferation. The consequences of Oct4 removal diverge from the consequences of removing another factor required for pluripotency between preimplantation development and early cell fate specification suggesting that the network Oct4 acts within is altered between these stages.

## Introduction

Oct4 is a homeodomain-containing transcription factor (TF) of the POU family required for pluripotency in ES cells and preimplantation embryos [1]. It has been extensively characterized in ES cells, and established as a hub of the signaling network that maintains pluripotency [2]–[5]. Embryonically, Oct4 is present in the developing zygote and down-regulated somatically between E7.0 and E9.0 depending on the cell type (see Supplementary (S) Figure (Fig.) S1 and S2 for detail) [6], [7]. After E9.0 of murine development Oct4 is restricted to the germline, persisting until maturation of type A to type B spermatogonia in the male germline, in contrast to the female gametic lineage where it is depleted during meiosis (E14–16) before up-regulation as oocytes mature within primordial follicles [6], [8]–[10]. Several regulators of Oct4 have been established *in vivo*. Oct4 is maintained through the early stages of embryonic development by intercellular Nodal acting in part through Smad2 [11], [12]. Conversely, Cdx2 mediates repression of Oct4 in trophectoderm of the early blastocyst, while both Eomes and Gcnf mediate repression in the embryo after implantation [13], [14].

Oct4 buffers the ICM against differentiation into trophectoderm (the embryonic contribution to the placenta), but the proposal that *Pou5f1* (gene symbol for Oct4) emergence relates to evolution of the mammalian placenta [15] is not supported given that *Pou5f1* evolved before the origin of amniotes [16]. It is unknown whether Oct4 has a conserved role, or any post-implantation function in murine somatic development. Pluripotent somatic cells persist until E7.5–8.5 based on teratogenesis experiments [17], [18] and ∼E8.0 based on epiblast stem cell (EpiSC) derivation [19], suggesting that Oct4 might continue to maintain pluripotency during this window of development. *in vitro* studies have also inferred many roles for Oct4 between the pre-streak and headfold stages, ∼E6.0–E8.0, including regulating neural versus mesendoderm differentiation [20], [21] as well as promoting cardiomyocyte [22] and neuronal differentiation [23]. However aside from maintaining the viability of primordial germ cells (PGCs), Oct4's role in post-implantation development has not been characterized *in vivo* [1], [2], [24], [25].

The extent of Oct4's function at the molecular level is also unclear. Physical interactions suggest Oct4 may have roles in chromatin modification, regulation of transcription, DNA replication and DNA repair as well as post-transcriptional modification, ubiquitination, and various other functions [2]–[4], [26], [27]. Oct4 both activates and represses transcription [28]. It binds thousands of sites in the ES cell genome, often co-occupying these sites with Sox2, Nanog, Smad1 and Stat3 [5]. The majority of genes occupied by several of these transcription factors (TFs) are active in ES cells, but their binding does not ensure expression [5].

Since Oct4 protein normally persists in somatic cells until ∼E7.0–E9.0 but *Pou5f1* null embryos arrest at E3.5, we asked what role Oct4 had later in murine development, using a conditional system to deplete it ∼E7.5. We show that Oct4 depletion ∼E7.5 results in craniorachischisis, random heart tube orientation, failed turning, defective somitogenesis as well as posterior truncation. The phenotype is not the result of a general delay in development, nor does it result from a failure in the pluripotency network present in the ICM. Depletion of Sox2, another core member of the pluripotency network in an overlapping window of development does not phenocopy Oct4 depletion. Instead, Oct4 is required until ∼E7.5 to maintain cell viability in the embryo and proliferation in the primitive streak. In its absence, convergent extension is disrupted leading to several morphogenetic defects.

## Results

### Oct4 is required for embryonic development until ∼E7.5

We used a conditional mutant of Oct4 to study its role after E3.5 when it is essential for development. We used floxed *Pou5f1* alleles (Oct4^f^) [25] and a tamoxifen inducible recombinase (CreER^T2^) that is ubiquitously expressed from the ROSA locus [29]. To establish the window of development during which embryos are sensitive to Oct4 depletion, we staggered the initial dose of tamoxifen with respect to embryonic maturity and administered a second supplementary dose 12 hrs later to enhance overall recombination efficiency. Oct4^f/f^;CreER^T2+/−^ embryos administered tamoxifen ∼E8.0 and ∼E8.5 before analysis ∼E9.5 did not have a phenotype (Table S1, row A (S1A), while tamoxifen administration ∼E7.5 and ∼E8.0 before analysis ∼E9.5 resulted in a partially penetrant phenotype (Fig. S3; Table S1B). Unlike tamoxifen administration beginning ∼E7.5 or ∼E8.0, all Oct4^f/f^; CreER^T2+/−^ embryos induced ∼E6.0 and ∼E6.5 before analysis ∼E9.5 were amorphous, lacking structures aside from what resembled anterior neural head folds (Fig. S4; Table S1C). Tamoxifen administration ∼E7.0 and ∼E7.5 also led to a fully penetrant phenotype ∼E9.5 (Table S1D).

**Figure 1:**
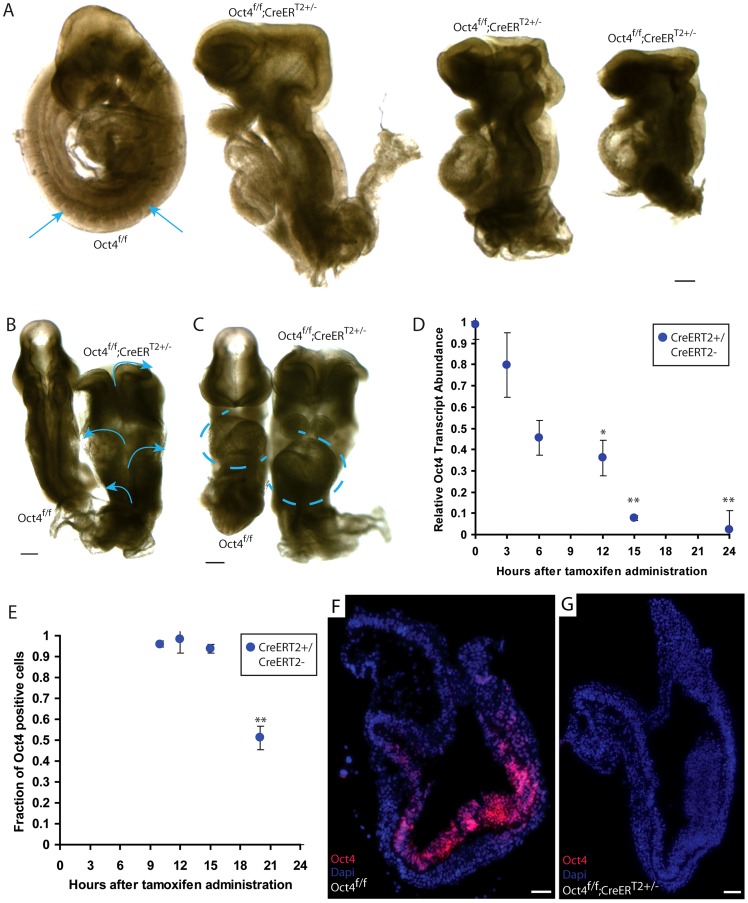
Ubiquitous depletion of Oct4 ∼E7.5 results in the Oct4^COND MUT^ phenotype ∼E9.5. **A–C** E9.5 Oct4^f/f^ embryo with no phenotype and Oct4^COND MUT^ (Oct4^f/f^;CreER^T2+/−^) littermates. Tamoxifen was administered ∼E7.0 and ∼E7.5 and embryos dissected ∼E9.5 (Table S1D). Scale bars in ‘A–C’ are 200 µm. **A** Sagittal view. Arrows indicate somites that are absent in the Oct4^COND MUT^ embryos. **B** Dorsal view. Arrows indicate the open neural tube. **C** Frontal view. Oct4^f/f^ with *situs solitus* (WT) heart tube orientation and Oct4^f/f^;CreER^T2+/−^ with *situs inversus* orientation. Heart tubes are outlined with dashed lines. **D** Relative transcript abundance (Oct4^f/f^;CreER^T2+/−^/Oct4^f/f^ littermates) measured using quantitative-PCR ± s.e.m. (inter-litter) indicates Oct4 transcript is significantly reduced 12 hrs ATA (F_5,13_ = 15.48, p<0.05 1-way ANOVA, *p<0.05, **p<0.01 Bonferroni posttest). **E** The fraction (Oct4^f/f^;CreER^T2+/−^/Oct4^f/f^ littermates) cells ± s.e.m. (intra-litter) with detectable Oct4 indicates a significantly different number of Oct4+ cells 20 hrs ATA (F_4,12_ = 51.86, p<0.05 1-way ANOVA, **p<0.01, ***p<0.001 Bonferroni posttest). **F,G** Oct4 (red) is depleted 24 hrs ATA. Nuclei are stained blue, anteriors are oriented to the left, and scale bars in ‘F,G’ are 50 µm.

E9.5 embryos administered tamoxifen ∼E7.0 and ∼E7.5 failed to turn, had severe posterior truncations, randomly oriented heart tubes, craniorachischisis (open neural tube along its entire length) as well as impaired somitogenesis (Fig. 1A–C). Such animals are referred to as Oct4^COND MUT^ in the remainder of this report. The phenotype is not a consequence of tamoxifen administration, leaky recombinase activity prior to tamoxifen administration, or associated with recombination of a single *Pou5f1* allele: no Oct4^f/f^ embryos induced ∼E7.0, no uninduced Oct4^f/f^;CreER^T2+/−^ embryos, nor any Oct4^+/f^;CreER^T2+/−^ embryos induced ∼E7.0 had phenotypes ∼E9.5 (Table S1E–G). Reducing the quantity of tamoxifen per dose administered ∼E7.0 or failure to administer the second dose ∼E7.5 led to incomplete penetrance of the Oct4^COND MUT^ phenotype (Table S1H–J): 80%, 40% and 0% of embryos ∼E9.5 exhibited the Oct4^COND MUT^ phenotype when a single full, half, and quarter tamoxifen dose was administered ∼E7.0 (Table S1H–J). This suggests reduced recombination with these lower tamoxifen doses. Collectively, these data support Oct4 depletion causing the Oct4^COND MUT^ phenotype.

To determine the time course of Oct4 depletion with this system, we compared Oct4 transcript and protein abundance between Oct4^f/f^ and Oct4^f/f^;CreER^T2+/−^ littermates administered tamoxifen ∼E7.0. A single dose of tamoxifen was used to avoid a compound effect from a second dose. Relative Oct4 transcript abundance (Oct4^f/f^;CreER^T2+/−^/Oct4^f/f^;CreER^T2−/−^ littermates) was significantly different 12 hrs after tamoxifen administration (ATA) (Fig. 1D; Table S1K; F_5,13_ = 15.48, p<0.05 1-way ANOVA, *p<0.05, **p<0.01 Bonferroni posttest). The fraction of cells in which Oct4 was detectable by immunohistochemistry was lower 20 hrs ATA, which is ∼E7.5 (Fig. 1E, Fig. S5A–D; Table S1L; F_3,10_ = 12, p<0.05 1-way ANOVA, **p<0.01 Bonferroni posttest). A distinct primary antibody indicated that Oct4 protein was undetectable 24 hrs ATA in Oct4^f/f^; CreER^T2+/−^ embryos (Fig. 1F,G; Table S1L). Since penetrance of the phenotype is complete when tamoxifen administration begins ∼E7.0, partial when tamoxifen administration begins ∼E7.5, and the fraction of cells with detectable Oct4 protein reduced ∼20 hrs ATA (following administration ∼E7.0), these data indicate that Oct4 is required until ∼E7.5.

### The Oct4^COND MUT^ phenotype

**Figure 2:**
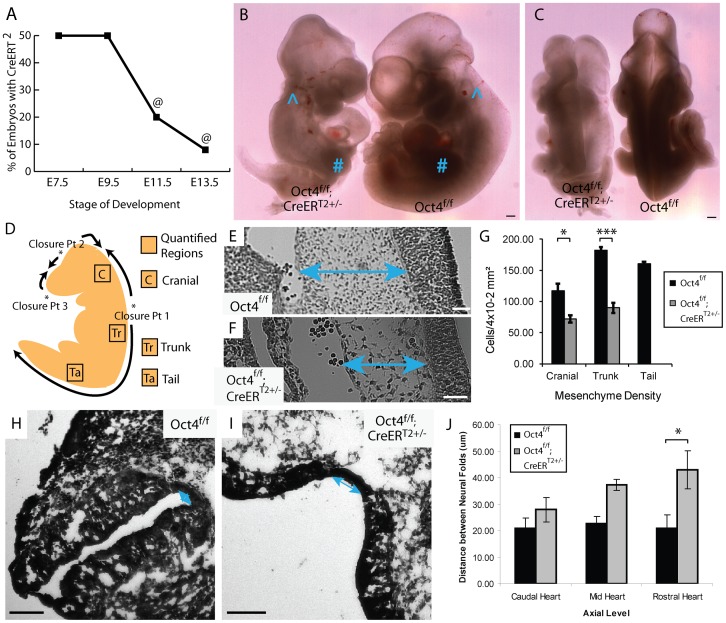
Depletion of Oct4 ∼E7.5 results in diminished viability, reduced mesenchyme density and broader spacing between the neural folds. **A** The fraction (Oct4^f/f^;CreER^T2+/−^/total) embryos recovered at each developmental stage (‘@’ indicates resorbing embryos). Embryos were administered tamoxifen ∼E7.0 and ∼E7.5 (Table S1M–O). **B,C** Representative images of an E10.5 Oct4^f/f^;CreER^T2+/−^ embryo and Oct4^f/f^ littermate following tamoxifen administration ∼E7.0 and ∼E7.5. Features such as the otic cup (∧) and forelimb bud (#) which normally arises after NTC is initiated and turning is complete are present in both Oct4^f/f^;CreER^T2+/−^ and Oct4^f/f^ littermates. Conversely, defects in turning, somitogenesis, neural tube closure and posterior extension all fail to recover in Oct4^f/f^;CreER^T2+/−^ embryos by E10.5. Scale bars in ‘B,C’ are 250 µm. **B** Sagittal view. **C** Dorsal view. **D** Schematic illustrating NT closure points, the directions in which the NT ‘zippers’ shut as well as the sections where mesenchyme density was quantified. **E,F** Representative Hematoxylin and Eosin (H and E) sections from the trunk (see ‘Tr’ in panel ‘D’) ∼E9.5. Differences in the density of mesenchyme, which is indicated with two-headed arrows, are apparent. Embryos were administered tamoxifen ∼E7.0 and ∼E7.5 and dissected ∼E9.5 (Table S1M–O). Scale bars in E,F are 50 µm. **E** Oct4^f/f^ **F** Oct4^f/f^;CreER^T2+/−^. **G** Mesenchyme, connective tissue comprised of mesoderm and neural crest cells, has a lower density in Oct4^COND MUT^. The average density within 4×10^−2^ mm^2^ ±s.e.m. (200 µm×200 µm) from sections equivalent to those in panel ‘E,F’ is plotted (F_1,13_ = 54.60, p<0.05 2-way ANOVA, *p<0.05, ***p<0.001 Bonferroni posttest). Due to the posterior truncation, insufficient mesenchyme was present in the tail of Oct4^COND MUT^ to quantify. **H–J** The neural tube in **H** Oct4^f/f^ **I** Oct4^f/f^;CreER^T2+/−^. Scale bars in ‘H,I’ are 100 µm. **J** The distance between neural folds in the trunk of Oct4^f/f^;CreER^T2+/−^ is broader (F_2,22_ = 17.42, p<0.05 2-way ANOVA, **p<0.01 Bonferroni posttest. Embryos in ‘H–J’ were administered tamoxifen ∼E7.0 and ∼E7.5 and dissected ∼E9.5 (Table S1M–O).

Oct4 depletion does not cause a global delay in development. Administering tamoxifen ∼E7.0 and ∼E7.5 to avoid partial penetrance, Oct4^f/f^;CreER^T2+/−^ embryos were recovered in a ratio of 1∶1 with Oct4^f/f^ littermates until E9.5, but less frequently at E11.5 (Fig. 2A; Table S1M–O). Features disrupted in Oct4^COND MUT^ remained arrested in the mutants that persisted beyond E9.5 (Fig. 2B,C), indicating that the Oct4^COND MUT^ phenotype is not a global delay in development but disruption of select features. Indentation of the otic cup occurred and the branchial arches formed in Oct4^COND MUT^, events that normally occur by E9.0. Forelimb buds also protruded in Oct4^COND MUT^ as they normally do by E9.5. Conversely, the neural tube normally closes rostrally between E8–9 and caudally by E9–10 (we refer to caudal and rostral neural tube closure with respect to closure point 1 at the hindbrain cervical boundary throughout; see Figure 2D) [30], turning normally occurs by ∼9.0 and posterior extension normally reaches 21–29 somites by E9.5 in WT embryos. These events always failed at E9.5 when *Pou5f1* excision was induced ∼E7.0 (Fig. 1A–C; Table S1D; 26.5 versus 4.6 somites in Oct4^f/f^ versus Oct4^f/f^;CreER^T2+/−^ littermates). Additionally, heart tube orientation was randomized, 38.6% of Oct4^f/f^;CreER^T2+/−^ had *situs inversus* while the orientation of 6.8% was ambiguous (Table S1P; p>0.05 Chi-square test). The neuroepithelium of Oct4^COND MUT^ embryos was also thicker in regions, particularly in the distal portion of the embryo (Fig. S6A–C; Table S1D; F_1,287_ = 94.95, p<0.05 2-way ANOVA, ***p<0.001 Bonferroni posttest). These data indicate that Oct4 is required for posterior extension, turning, heart tube orientation and neural tube closure (NTC).

Partial phenotype penetrance following tamoxifen administration ∼E7.5 was used to assess whether the cause of disrupted features in Oct4^COND MUT^ embryos were related. Coincidence of features in litters with incomplete phenotype penetrance suggests related causation of the coincident features. Craniorachischisis and posterior truncation coincided in all 23 of the 36 embryos analyzed (Fig. S2; Table S1B; p = 1.64E-10, hypergeometric test). Conversely 2 turning defects in the 9 embryos where rostral NTC failed suggests independence of these processes, although the small number of embryos limits statistical power in this case (Fig. S3; Table S1B; p = 0.72, hypergeometric test). These data suggest independent requirements for Oct4 in closure at closure point 1/posterior extension and rostral NTC.

Craniorachischisis occurs when closure at closure point 1 fails (see Figure 2D). Convergent extension elongates the embryo in the anterior-posterior axis during gastrulation and neurulation, bringing the neural folds into opposition prior to adhesion at closure point 1. Failed convergent extension results in broad midlines and enlarged notochord diameter as both narrow during convergent extension. Oct4^COND MUT^ embryos exhibit broad neural plates (Fig. 2H–J; Table S1D; F_2,22_ = 17.42, p<0.05 2-way ANOVA, **p<0.01 Bonferroni posttest) and enlarged notochord diameter (Fig. S6D–F; Table S1D; p<0.05, two-tailed student t-test). Concordance between posterior truncation and craniorachischisis, broadened neural plates, and broader notochords are consistent with deficient convergent extension.

NTC rostral and caudal to closure point 1 occur by different mechanisms. Unlike the spinal region where expansion of paraxial mesoderm is not required for elevation and subsequent NTC, cranial NTC is initiated by expansion of underlying mesenchyme [30]. Mesenchyme density, including cranial mesenchyme, was reduced in Oct4^COND MUT^ (Fig. 2E–G; Table S1D; F_1,13_ = 54.59, p<0.05 2-way ANOVA, *p<0.05, ***p<0.001 Bonferroni posttest). Hence expansion of cranial mesenchyme that is required for cranial NTC is deficient in the absence of Oct4.

### Extraembryonic Oct4 is not required ∼E7.5

**Figure 3:**
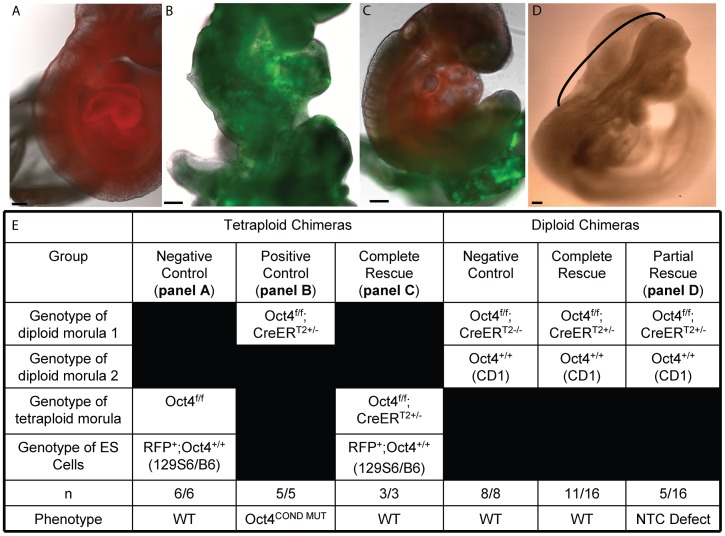
Tetraploid chimeras indicate that Oct4 is not required extraembryonically ∼E7.5, while diploid chimeras indicate that Oct4^+/+^ can compensate for Oct4^−/−^ cells embryonically. All embryos transferred to a surrogate and depicted or described in panels A–E were induced with tamoxifen ∼E6.0 and ∼E6.5 to compensate for the variability in developmental timing associated with transfer (Table S1Q,AB). Scale bars in panels A–D are 200 um. **A** A representative embryo from aggregation of Oct4^+/+^ RFP ES cells with a tetraploid Oct4^f/f^ embryo. Oct4^f/f^ extraembryonic tissue yielded E9.5 chimeric embryos with no phenotype. **B** An Oct4^f/f^;CreER^T2+/−^ E9.5 embryo with the Oct4^COND MUT^ phenotype. **C** Oct4 depletion in extraembryonic tissue is compatible with WT development. A representative chimera consisting of RFP+ Oct4^+/+^ ES cell derived embryo and tetraploid Oct4^f/f^;CreER^T2+/−^ extraembryonic tissue. The embryo has turned (compare panel ‘B’ where the tail is behind to panel ‘C’ where it is in front), undergone NTC and posterior extension (compare the lack of somites and short tail in panel ‘B’ to the somites and full-length tail in ‘C’). **D** The most severe embryonic defect observed in a diploid chimeras consisting of Oct4^+/+^ and Oct4^f/f^;CreER^T2+/−^ cells. The neural tube is open between closure points 1 and 2, indicated here with a black bracket. All Oct4^COND MUT^ features aside from cranial NTC defect, which is still present in 5/16 mosaic embryos, are rescued by Oct4^+/+^ cells in these diploid chimeras (16/16). For example, this embryo has ‘turned’ such that it faces its tail and the posterior has extended normally. **E** Quantification of the genotypes and phenotypes of recovered chimeric embryos.

A requirement for Oct4 in extraembryonic tissue offers one possible explanation for the Oct4^COND MUT^ phenotype: ∼E7.5 Oct4 is present in extraembryonic mesoderm, allantoic angioblasts as well as extraembryonic endoderm which promotes proliferation and organization of the primitive streak [6], [31]. To test this possibility, Oct4^+/+^ Red fluorescent protein positive (RFP^+^) ES cells were aggregated with tetraploid Oct4^f/f^;Z/EG^+/−^;CreER^T2+/−^ embryos, where ES cells contribute to the embryo, and tetraploid cells generate trophectoderm and visceral endoderm [32]. In this scheme, tamoxifen administration will selectively remove of Oct4 from the tetraploid extraembryonic lineages. Tetraploid Oct4^f/f^;Z/EG^+/−^;CreER^T2+/−^ embryos induced ∼E6.5 and ∼E7.0 supported development of WT ES-derived embryos to E9.5 (Fig. 3A–C,E; Table S1Q). Embryos were dosed on this relatively early schedule to avoid false negatives that might result from altered timing of development associated with transferring embryos to pseudopregnant mothers. In practice transferred embryos synchronize with the maternal uterine environment [33], suggesting false negatives for this reason are unlikely. Normal embryonic development after excision of *Pou5f1* in trophectoderm and visceral endoderm suggests Oct4 is required in embryonic tissue.

### Mosaic Oct4 depletion is compensated by WT cells

To identify non-autonomous effects of Oct4 depletion, we tested whether lineage-specific removal of Oct4 affected development of other tissues. Since Oct4 is present in the primitive streak, neuroepithelium and portions of mesoderm ∼E7.5 as well as mosaically in definitive endoderm (Fig. S1 and S2), a primary effect in one of these lineages might non-autonomously cause other aspects of the Oct4^COND MUT^ phenotype [6]. To test this possibility, Oct4 was removed in the neuroepithelium using Sox1-Cre, which is expressed and catalytically active from ∼E7.5 [34]; in definitive endoderm using tamoxifen-inducible Foxa2^mcm^, which is expressed ∼E6.25 [35]; as well as in embryonic mesoderm using Brachyury (Bry)-Cre, which is expressed and catalytically active from ∼E6.25 [36].

Excision of *Pou5f1* by lineage-specific recombinases (Bry-Cre, Sox1-Cre or Foxa2^mcm^) did not result in a phenotype or impact embryonic viability at E9.5. Oct4^f/f^; Z/EG^+/^; lineage-specific Cre^+/−^ embryos should reveal aspects of the Oct4^COND MUT^ phenotype related to requirements for Oct4 within their respective expression domains or cause the embryo to resorb by E9.5 if development is more severely impacted than in Oct4^COND MUT^ embryos. Recombination at the lacZ/enhanced GFP (Z/EG) locus yields GFP expression, so the Z/EG allele was incorporated to gauge recombination efficiency [37]. Based on the parental genotypes used in the cross (Table S1R–T), a genotypic ratio where Oct4^f/f^; Z/EG^+/−^; lineage-specific Cre^+/−^ embryos comprise ¼ of the progeny is expected if this genotype, where lineage-specific excision of *Pou5f1* occurs, does not impact viability. Such embryos with no phenotype comprised ¼ of each litter (Table S1R–T). To test whether the lineage-specific recombinases yielded false negative results due to infrequent biallelic excision, we assessed the development of embryos where one *Pou5f1* allele was removed prior to recombinase expression. Even with this sensitized approach, Oct4^Δ/f^; Z/EG^+/−^; lineage-specific Cre^+/−^ embryos with no phenotype comprised ¼ of the progeny at E9.5. This genotypic ratio indicates that excision of *Pou5f1* by these lineage-specific recombinases did not impact viability (Table S1U,V).

Since false-negatives may arise due to low recombination efficiency in this scheme, we used the GFP expression resulting from recombination at the Z/EG locus in Oct4^f/+^; Z/EG^+/−^; lineage-specific Cre^+/−^ embryos as a proxy for recombination efficiency. By E9.0 Sox1-Cre and Bry1-Cre induced >95% and >51% recombination within their respective domains (Fig. S7A–C; Table S1W–Y), while Foxa2^mcm^ yielded <5% (data not shown). However, prior to E8.0 when embryos are sensitive to Oct4 depletion, Sox1-Cre and Bry-Cre also yielded <5% recombination (Fig. S7C; Table S1Z,AA) [30]. Notably, the distribution of Oct4^Δ/f^; Z/EG^+/−^; Bry-Cre^+/−^ cells did not appear altered ∼E9.5 (Fig. S7D,E), suggesting that any effect Oct4 has on cell fate either coincides with lineage specification or precedes it.

To investigate how recombination frequency influences phenotype penetrance in embryos where *Pou5f1* is removed by lineage-specific recombinases, we generated diploid chimeras by aggregating WT and Oct4^f/f^;HisGFP^+/−^;CreER^T2+/−^ morulas. The ubiquitously expressed fusion protein ‘HisGFP,’ which is comprised of histone H2B and eGFP was used to mark transgenic cells [38]. Following tamoxifen administration ∼E6.5 and ∼E7.0, we recovered 16 chimeras where contribution by Oct4^f/f^;HisGFP^+/−^;CreER^T2+/−^ morulas ranged from 20–60% (Table S1AB). 11 of these 16 embryos had no phenotype, while the remaining 5 chimeras had rostral NTC deficits (Fig. 3D,E). This indicates that Oct4^+/+^ cells rescue the developmental deficiencies caused by Oct4^−/−^ cells in mosaic embryos. Since efficient depletion of Oct4 is required for the Oct4^COND MUT^ phenotype, the inefficient recombination of Bry-Cre, Sox1-Cre and Foxa2^mcm^ during the window of development in which embryos are sensitive to Oct4 depletion does not resolve whether Oct4 is ubiquitously required ∼E7.5, required only in unspecified progenitors, or necessary in a subset of specified lineages, such as in specified Oct4+Bry+ mesoderm.

Since this data suggested that differences in the kinetics of *Pou5f1* excision with lineage-specific recombinases and CreER^T2^ (when tamoxifen is administered ∼E7.0) are responsible for the absence and presence of phenotypes following *Pou5f1* excision, we tested whether expansion of specified lineages was affected in Oct4^COND MUT^ embryos. Lineage-specified Bry+ and Sox2+ cells were present 48 hrs ATA in Oct4^f/f^;CreER^T2+/−^ embryos (Fig. S8A,B; Table S1AC). We quantified the fraction of phosphorylated Histone H3 (PH3)+ cells in specified lineages. The PH3+ fraction of neural or mesoderm cells (Oct4^f/f^;CreER^T2+/−^ versus Oct4^f/f^) was the same (Fig. S8C, Table S1AC). The data indicate that expansion of these specified lineages is not impacted by Oct4 depletion.

### Depletion of Oct4 and Sox2 ∼E7.5 do not phenocopy

**Figure 4:**
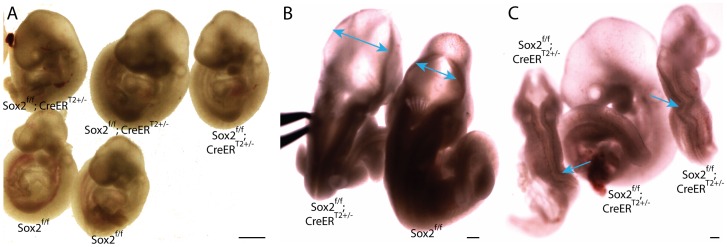
Depletion of pluripotency factor Sox2 does not phenocopy Oct4 depletion ∼E7.5. **A–C** Sox2^f/f^;CreER^T2+/−^ embryos were administered tamoxifen ∼E6.5 and ∼E7.0 and dissected ∼E9.5 (Table S1AD). **A** Sagittal view of Sox2^f/f^;CreER^T2+/−^ and Sox2^f/f^ littermates. 11/20 Sox2^f/f^;CreER^T2+/−^ embryos had hydrocephalus ∼E9.5. **B** Dorsal view of a Sox2^f/f^;CreER^T2+/−^ embryo with hydrocephalus. Two-headed arrows indicate region where neuroepithelium does not approach the midline of Sox2^f/f^;CreER^T2+/−^ as it does in Sox2^f/f^ embryos. **C** 2/20 Sox2^f/f^;CreER^T2+/−^ had kinked neural tubes (the kinking is indicated with arrows) without hydrocephalus (at left and right), while the embryo in the middle has the more prevalent hydrocephalus. Neither hydrocephalus nor kinked neural tubes were observed in Oct4^COND MUT^ embryos.

To test whether disruption of the pluripotency network causes the Oct4^COND MUT^ phenotype, we removed *Sox2* using the same conditional approach [39]. Sox2 is a core component of the pluripotency network that complexes with Oct4, co-occupies many genomic sites (Oct4/Sox2) and is required for maintenance of *Pou5f1* expression in ES cells. ES cells differentiate into trophectoderm when *Sox2* is removed [40], however the ability of Oct4 over-expression to rescue pluripotency in these cells suggests that the critical role of Sox2 in pluripotency is to maintain *Pou5f1* expression [40]. *Sox2* null embryos lack epithelial cells typical of the epiblast and have a later extraembryonic defect which does not permit development past E7.5 [41]. Following tamoxifen administration ∼E6.5 and ∼E7.0 to Sox2^f/f^;CreER^T2+/−^ embryos [39], hydrocephalus was evident in 11/20 Sox2^f/f^;CreER^T2+/−^ and 2/20 others had kinked neural tubes ∼E9.5 (Fig. 4A–C; Table S1AD). Thus Sox2 removal did not phenocopy Oct4 depletion ∼E7.5. These data do not rule out partial compensation for loss of Sox2 by redundant factors, however between E7.0–E8.0 Oct4 and Sox2 only overlap spatially in anterior neuroepithelium (compare Figure S1, S2 and S9) [6], [41]. The distinct phenotypes produced by depletion of *Sox2* and *Pou5f1* indicate that at least part of their functions do not overlap ∼E7.0–E8.0, in contrast to ES cells.

### Oct4 acts as a repressor, and at sites co-occupied by Sox2, an activator, ∼E7.5–E8.5

Oct4 is reported to bind 784–4234 genomic loci in ES cells depending on the methodology used to map binding sites [5], [42], [43]. To determine which targets might be contributing to the Oct4^COND MUT^ phenotype, we measured gene expression changes that occurred coincident with Oct4 depletion (∼E7.5) and thereafter (∼E8.0 and ∼E8.5). Oct4^f/f^;CreER^T2+/−^ embryos were separated from Oct4^f/f^ littermates by genotyping extraembryonic tissue, and differential expression assessed within litters with ≥3 CreER^T2+/−^ and ≥3 CreER^T2−/−^ embryos (Table S1AE). RNA was extracted 24, 36 and 48 hrs ATA, when Oct4 transcript abundance in CreER^T2+/−^ embryos is <5% CreER^T2−/−^ littermates (Fig. S5A–D). 754 unique genes were differentially expressed (p<0.01) at one or more of these three timepoints.

To determine whether the differential expression following Oct4 depletion was a direct consequence of Oct4 loss at its genomic targets, we assessed whether Oct4's direct targets were enriched amongst up- or down-regulated genes as Oct4 both activates and represses transcription [28]. Systematic mapping of TF targets in early embryos is currently prohibitive [44], so a genome-wide binding map of Oct4 in ES cells was used [5]. This particular genomic binding map, which is based on ChIP-seq data, was used because it offers more complete genomic coverage than target maps based on ChIP-chip data, and also contained the most extensive set of other TF binding maps for additional analysis (alternatives include: [42], [43]).

**Figure 5:**
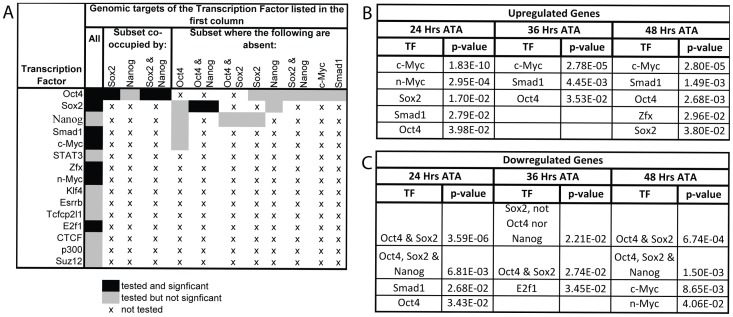
Gene expression profiling coincident with and following Oct4 depletion indicates that c-Myc, Smad1 and Oct4 targets are up-regulated while targets co-occupied by Oct4 and Sox2 are down-regulated. **A** Combinations of TFs whose target sets were tested for enrichment amongst differentially expressed genes. **B,C** After the loss of Oct4 up-regulated genes are consistently enriched for targets of c-Myc, Smad1 and Oct4 while down-regulated genes are enriched for targets bound by both Oct4 and Sox2. Litters were induced with tamoxifen ∼E7.0 (Table S1AE). The FDR for reported enrichments in ‘B,C’ is <0.001, based on 1000 random permutations of annotated genes. **B** TF binding enrichment amongst up-regulated genes using hypergeometric tests. **C** TF binding enrichment amongst down-regulated genes using hypergeometric tests.

Enrichment of TF binding targets from ES cells amongst differentially expressed genes after ∼E7.5 requires that binding sites be conserved between these stages. Oct4 binding sites from ES cells were enriched amongst up-regulated genes (Fig. 5B), supporting conservation of the binding sites between ES and ∼E7.5–E8.5 embryos. Oct4 binding targets were also enriched when alternative datasets were analyzed. For comparison, with the aggregate of differentially expressed genes (24, 36 and 48 hrs ATA), enrichment using hypergeometric tests were: p = 3.45E-11 [43], p = 2.13E-08 [5], and p = 7.36E-4 [42]. This suggest that the expression changes at these sites were a direct consequence of Oct4-mediated transcriptional regulation being removed after ∼E7.5.

Oct4 targets whose transcription is regulated by Oct4 in ES cells were differentially expressed coincident with Oct4 depletion ∼E7.5. *Lefty1* and *Klf2* that are activated by Oct4 in ES cells decreased [45], [46], while *Xist* was notable among the most up-regulated genes following Oct4 depletion as it is repressed by Oct4 in ES cells [41]. An unbalanced male∶female ratio in the intra-litter comparisons, rather than Oct4 depletion, might explain the increase in *Xist* transcript abundance since embryos were not sexed in the microarray, however Quantitative (Q)-PCR on independent balanced comparisons confirmed that the increase related to Oct4 depletion. An intra-litter comparisons to match developmental stage, and inter-litter comparisons to reduce biological variance associated with comparing a small number of embryos both supported Oct4-mediated repression of *Xist* ∼E7.5: *Xist* was 3.20 times more abundant in the intra-litter comparison, and 2.85±0.76 s.e.m. more abundant in the inter-litter comparison of Oct4^f/f^;CreER^T2+/−^/Oct4^f/f^ 24 hrs ATA (Table S1AF).

Enrichment for genomic targets of Oct4 is expected with this approach, but transcriptional activators of Oct4 and proteins that physically interact with it were also differentially expressed. Ligands that maintain Oct4 such as *Nodal* and *Wnt3a* [11], [47] exhibit decreased transcript abundance coincident with Oct4 depletion ∼E7.5, while transcriptional activators of Oct4 such as *Sp1* [48] and *Ago2* [49] exhibited increased transcript abundance, perhaps due to a feedback loop. Proteins that physically interact with Oct4 were also enriched amongst the genes up-regulated following Oct4 depletion (see Table S2 for cofactor identities; p = 1.99E-08 24 hrs ATA, p = 1.64E-05 36 hrs ATA, p = 5.55E-07 48 hrs ATA enrichment using hypergeometric tests). Interestingly, we found considerable enrichment for Oct4 within genomic regulatory elements of these physical cofactors (p = 5.34E-07 for 24,36 and 48 hrs ATA collectively using a hypergeometric test). This suggests that ∼E7.5 Oct4 directly represses expression of a subset of the genes it physically interacts with in ES cells and that its absence triggers positive indirect feedback of the expression of others. Collectively, these data suggest that several regulatory relationships of Oct4 are maintained between preimplantation development and ∼E7.5–8.5.

To test whether signaling networks other than direct targets of Oct4 might contribute to the Oct4^COND MUT^ phenotype, we determined the transcriptional response that target sets bound by TFs other than Oct4 had to Oct4 depletion. The binding maps of 12 other TFs, and combination of several with Oct4, were assessed for enrichment amongst the genes differentially expressed after Oct4 depletion (Fig. 5A) [5]. Targets of c-Myc and Smad1 were enriched amongst genes up-regulated after Oct4 depletion [5]. Unlike c-Myc, which does not cluster at binding sites with Oct4 in the genome, Oct4 facilitates the binding of Smad1 such that they overlap at a subset of sites [5]. However up-regulation of Smad1 targets after Oct4 depletion occurred at sites Smad1 occupies independent of Oct4, indicating that enrichment of up-regulated Smad1 targets is not due to direct relief of Oct4-mediated repression at sites that the two co-occupy [5]. The enrichment of Smad1 targets amongst up-regulated genes that are not co-occupied by Oct4 are: p = 6.14E-06 24 hr ATA, p = 4.55E-03 36 hr ATA, p = 3.53E-09 48 hr ATA (hypergeometric test). Like Oct4, Smad1 has been implicated in both activation and repression of target genes [50], consistent with a separate subset of Smad1 targets are de-repressed 24 hrs ATA. These data suggest that the absence of Oct4 yields a transcriptional environment conducive to target activation by c-Myc and Smad1. Conversely, enrichment of co-occupied Oct4/Sox2 target sites amongst down-regulated genes (Fig. 5C) suggests that Oct4 participates in transcriptional activation of these ∼E7.5 and after. Since conditional removal of *Sox2* and *Pou5f1* do not phenocopy (compare Figure 1A to 4A), Sox2 is either not essential for activation of these sites, which is consistent with data from ES cells [40], or down-regulation of these targets does not contribute to the Oct4^COND MUT^ phenotype.

### Primary transcriptional responses following Oct4 depletion

**Figure 6:**
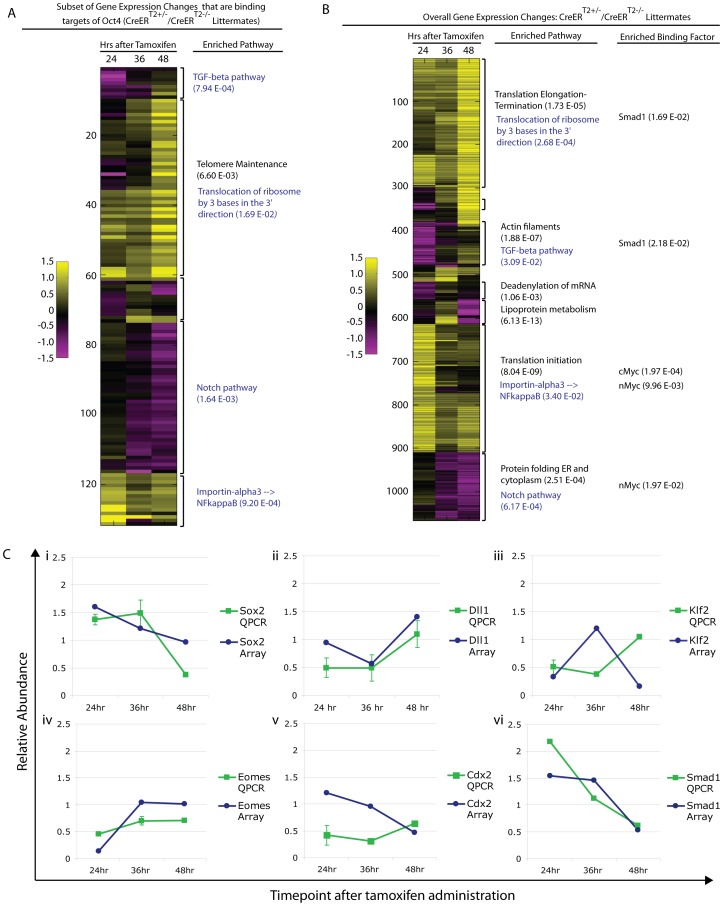
Pathway enrichment and confirmation of a subset of differentially expressed genes following Oct4 depletion. Litters depicted in ‘A,B’ were induced with tamoxifen ∼E7.0 (Table S1AE). The FDR for reported enrichments in ‘A,B’ is <0.001, based on 1000 random permutations of annotated genes. **A** Unsupervised clustering of relative (Oct4^f/f^;CreER^T2+/−^/Oct4^f/f^ littermates) gene expression sub-setted for Oct4 binding targets following Oct4 depletion. Enrichment for the same pathways in the global differential expression set and subset directly targeted by Oct4 support the utility of sub-setting for Oct4 binding targets in identifying primary effects of Oct4 depletion and the relevance of these primary effects to the Oct4^COND MUT^ phenotype in that they appear amplified into effects on the overall gene expression profile (see blue script in panel ‘A’ and ‘B’ for these). The most enriched pathway is provided for each cluster, and an additional pathway provided (in black text) for the cluster where the most enriched pathway in the Oct4 target set did not translate to a global change. **B** Unsupervised clustering of global differential expression (same dataset as panel ‘A’): Oct4^f/f^;CreER^T2+/−^/Oct4^f/f^. The most enriched pathway and binding factor are provided for each cluster (black text), while primary effects that translated to enriched effects in the global set are in blue text. **C** Confirmation of expression change for select genes by quantitative PCR in independent litters (Oct4^f/f^;CreER^T2+/−^/Oct4^f/f^ ±s.e.m.). Litters were induced with tamoxifen ∼E7.0 (Table S1AG).

Oct4 binds thousands of sites in the genome, and it is unlikely that disruption of a single target gene causes the Oct4^COND MUT^ phenotype. To relate molecular changes resulting from Oct4 depletion with the Oct4^COND MUT^ phenotype, we determined which signaling pathways were disrupted coincident with Oct4 depletion and prior to the onset of the phenotype. Unsupervised clustering was used to assess the function of differentially expressed genes collectively. To discern primary effects of Oct4 depletion, we sub-setted for genes that are direct targets of Oct4 based on the ES binding maps [5], clustered these (Fig. 6A; Table S1AE), and then compared the clusters to global changes (Fig. 6B; Table S1AE). 3 of the 4 pathways showing the strongest enrichment in the set of direct targets also showed significant enrichment in the global set. Coordinate regulation of additional genes that are not targets of Oct4 within the same pathways as those directly regulated by Oct4, suggests amplification of the direct effects (Fig. 6A,B; Table S1AE). QPCR on independent biological samples confirmed a subset of changes from the global expression analysis (Fig. 6C, Table S1AG), supporting the reproducibility of the differential expression.

Differential expression was then considered in relation to the Oct4^COND MUT^ phenotype. The expression profiling suggested that decreased TGF-β signaling and increased nuclear import of NF-κB were primary effects as they occurred within hours of Oct4 depletion (24 hrs ATA) amongst direct targets of Oct4, while decreased Notch signaling and increased protein translation are other candidates that occurred later (Fig. 5A).

**Figure 7:**
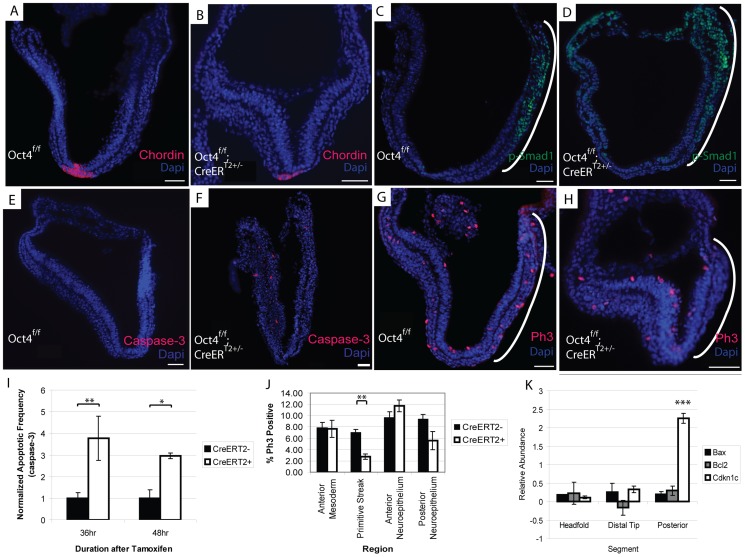
Decreased proliferation in the primitive streak occurs coincident with Oct4 depletion. The embryos depicted in panels ‘A–H’ were administered tamoxifen ∼E7.0 and ∼E7.5 and dissected 24 hrs ATA (Table S1AC). Scale bars in ‘A–H’ are 50 µm. **A, B** Specification of Chordin in the node still occurs after Oct4 depletion **A** Oct4^f/f^ 24 hrs ATA. **B** Oct4^f/f^;CreER^T2+/−^ 24 hrs ATA. **C, D** The expression domain of p-Smad1 is altered after Oct4 depletion. **C** Oct4^f/f^ 24 hrs ATA **D** Oct4^f/f^;CreER^T2+/−^ 24 hrs ATA. **E,F** Distribution of apoptotic Caspase-3+ cells 24 hrs ATA. **E** Oct4^f/f^ **F** Oct4^f/f^;CreER^T2+/−^. **G,H** Distribution of Phospho-histone-3 (Ph3), which marks proliferating cells, 24 hrs ATA. Proliferation is significantly reduced in the primitive streak (bracketed by a white line) of Oct4^f/f^;CreER^T2+/−^ embryos. (G) Oct4^f/f^ (H) Oct4^f/f^;CreER^T2+/−^. **I** Quantification of apoptotic frequency ±s.e.m. in Oct4^f/f^ and Oct4^f/f^;CreER^T2+/−^ embryos (F_1,44_ = 13.16, p<0.05 2-way ANOVA, *p<0.05, **p<0.01 Bonferroni posttest). **J** Quantification of proliferation frequency ±s.e.m. in Oct4^f/f^ and Oct4^f/f^;CreER^T2+/−^ embryos 24 hrs ATA (F_1,68_ = 3.28, p<0.05 2-way ANOVA, **p<0.01 Bonferroni posttest). Oct4 removal only effects proliferation significantly in the primitive streak. **K** Distribution of gene expression changes in Oct4^f/f^;CreER^T2+/−^ embryos. Relative (Oct4^f/f^;CreER^T2+/−^/Oct4^f/f^) transcript abundance ±s.e.m. was quantified 24 hrs ATA by QPCR (F_2,8_ = 12.14, p<0.05 2-way ANOVA, ***p<0.001 Bonferroni posttest). Embryos were administered tamoxifen ∼E7.0 and ∼E7.5 and dissected 24 hrs ATA for the experiment in panel ‘K’ (Table S1AH).

The node is required to coordinate left-right asymmetry, specification of definitive endoderm and somitogenesis [51]. Given these roles in development, we considered the possibility that Oct4 was required in node formation a candidate that might explain the *situs inversus*, defective somitogenesis and the posterior truncation (via either endoderm specification or defective somitogenesis) observed in Oct4^COND MUT^ embryos. Gene expression changes following Oct4 depletion also suggested the possibility of node malformation: decreased Dll1 contributed to the ‘Notch signaling’ enrichment in the microarray and was confirmed by QPCR in separate litters (Fig. 6C; Table S1AG). Decreased Dll1 following Oct4 depletion is relevant because loss of Dll1 was previously shown to disrupt node formation and cause defects in left/right asymmetry [52]. While these data were suggestive of a candidate mechanism underlying the Oct4^COND MUT^ phenotype, the presence and appropriate localization of the node marker Chordin both 24 hrs ATA (Fig. 7A,B, Table S1AC) and 36 hrs ATA (Fig. S10A,B; Table S1AC) suggests that initial node specification occurs in Oct4^COND MUT^ [53]. The disruption of left-right asymmetry is likely downstream of node specification, as transcript abundance of laterality specifiers that are asymmetrically distributed by the node during development is altered: *Nodal*, *Dll1*, *Lefty1* and *Lefty2* are decreased while *Hand1* and *Hand2* are increased. These data do not support the Oct4^COND MUT^ phenotype being caused by a failure in Notch-mediated node specification.

Contraction of actin-myosin microfilaments contributes to the morphogenetic processes of turning and convergent extension. A decrease in ‘actin filaments’ (p = 1.88E-07) following Oct4 depletion (Fig. 6B; Table S1AE) suggests that actin networks are affected by Oct4 depletion. The distribution of actin appeared altered 24 hrs ATA with phalloidin staining (Fig. S10C,D; Table S1AC). Indeed the distribution of actin in Oct4^f/f^;CreER^T2+/−^ embryos suggests that adhesion between anterior and posterior neuroepithelium in the distal portion of the embryo may contribute to thicker neuroepithelium in this regions and impaired embryonic morphogenesis.

TGF-β signaling has also been implicated in several processes disrupted in Oct4^COND MUT^ embryos: expansion of primitive streak [54], patterning derivatives of the anterior primitive streak [55], establishment of definitive endoderm [56], maturation of the node [57] and left/right asymmetry establishment [58], [59]. Unsupervised clustering indicates that Oct4 directly maintains TGF-β signaling (Fig. 6A). TGF-β signaling through Smad2 competes with Smad1 for the co-activator Smad4 [60], so up-regulation of Smad1 targets following Oct4 depletion may involve an increase in Smad1, expansion of the domain of activated phosphorylated-Smad1 (p-Smad1), or diminished competition from TGF-β-Smad2. Increased transcript abundance of Smad1 was confirmed by Q-PCR (Fig. 5C; Table S1AG). The p-Smad1 domain also appears altered 24 hrs ATA (Fig. 7C,D; Table S1AC). Variance in p-Smad1 introduced by differences in embryonic stage and ‘batch effects’ during detection prohibited making a statistically meaningful quantitative comparison of protein abundance between stage-matched Oct4^f/f^; CreER^T2+/−^ and Oct4^f/f^ embryos. Quantitative comparison with high-content image analysis software did suggest a difference in p-Smad1 abundance related to Oct4 depletion (Fig. S11), but this approach would require a considerable increase in sample size to test significance. These data suggest a direct effect of Oct4 depletion on diminished TGF-β signaling.

### Oct4 depletion leads to decreased proliferation in the primitive streak

Presence of Oct4 in the primitive streak ∼E7.5 (Fig. S1), impaired axial extension in Oct4^COND MUT^ embryos and differential expression of TGF-β signaling that is essential for expansion of primitive streak [54] suggested an effect on its expansion. An effect on the primitive streak and consequently its derivatives might have broad relevance: cranial mesenchyme supports NTC, while mesendoderm facilitates posterior extension, somitogenesis and turning. The frequency of cells undergoing apoptosis (Caspase-3+) in the Oct4^COND MUT^ was increased (Fig. 7I; Table S1AC), suggesting that diminished cell viability might contribute to the phenotype. Notably, the distribution of apoptotic cells throughout the embryo, including regions where Oct4 is not expressed, suggests that some apoptosis may be a secondary defect. Conversely, fewer cells proliferated indicated by phosphorylated histone H3 positive (PH3+) in the primitive streak of embryos 24 hrs ATA (Fig. 7G,H,J; Table S1AC). To confirm the localization of these effects, we divided embryos into three segments (proximal anterior, distal and proximal posterior) and quantified the abundance of transcripts regulating apoptosis and proliferation. To obtain sufficient material for comparison, CreER^T2+/−^;Oct4^f/f^ samples 24 hrs ATA were compared to CreER^T2+/−^;Oct4^f/f^ stage-matched samples from separate litters. While there was no difference in the transcript abundance of apoptosis regulators *Bax* and *Bcl2*, a negative regulator of proliferation, *Cdkn1c*, which exhibited increased transcript abundance in the differential expression analysis was selectively increased in the posterior third of embryos coincident with the loss of Oct4 (Fig. 7K; Table S1AH). These data indicate that ubiquitous Oct4 depletion leads to increased apoptosis and deficient proliferation in the primitive streak.

## Discussion

∼E7.5, Oct4 is still present in the primitive streak, posterior visceral endoderm, several mesoderm derivatives, neuroepithelium as well as extraembryonic endoderm and mesoderm (Fig. S1) [6]. Proliferation of the primitive streak decreases and apoptosis increases within the embryo coincident with Oct4 depletion ∼E7.5, and by ∼E9.5 several morphogenetic processes are disrupted: turning, posterior extension, laterality and NTC all are affected, demonstrating that Oct4 is required for somatic development after implantation.

Reduced proliferation in the primitive streak coincident with Oct4 depletion suggests that Oct4 might maintain potency ∼E7.5 as it does in the ICM [1]. EpiSC-derivation and teratoma assays support the persistence of pluripotent somatic cells ∼E8.0, while lineage tracing indicates the presence of neuro-mesodermal progenitors ∼E8.0 [61]. However excision of pluripotency factors Sox2 and Oct4 ∼E7.0 do not phenocopy as their depletion in ES cells do [1], [40], indicating that the pluripotency network is altered between the ICM and ∼E7.5. Differences in localization contribute: at the latest stage embryos are sensitive to Oct4 depletion and a proliferation deficit is evident in the primitive streak of Oct4^COND MUT^ embryos (∼E7.5), Sox2 transcript is limited to the chorion and anterior neuroectoderm (Fig. S9) [41]. Neural-specific Sox2 excision results in enlarged lateral ventricles ∼E19.5 due to decreased proliferation of neural stem and progenitor cells [62], suggesting that hydrocephalus in Sox2^COND MUT^ embryos may result from insufficient expansion/thickening of the neuroepithelium. This might render the neuroepithelium more elastic and distended as a result of the positive fluid pressure in the neural lumen [63], or precede the collapse or kinking of neural tubes that infrequently occurred. The differing phenotypes following depletion ∼E7.5 indicate that Sox2 is not required for *Pou5f1* transcription or as a cofactor in the processes disrupted in Oct4^COND MUT^ embryos.

Oct4 promotes mesoderm as opposed to neural fate during ES differentiation [20], as does *XlPou91* (the paralog in *X. laevis*) in response to FGF [64], [65], suggesting that Oct4 depletion might divert mesoderm to neural tissue. Decreased expression of *Tbx6* [66] and *Wnt3a* [67] whose loss is associated with diversion to ectopic neural tubes from paraxial mesoderm following Oct4 depletion is consistent with this possibility, as is thicker neuroepithelium of Oct4^COND MUT^ embryos near closure point 1. However this differential expression may not reflect altered specification *per se*, but altered proportions of the embryo associated with defective axial extension. Similarly, neuroepithelial thickening unrelated to cell fate divergence is common amongst mutants with NTC defects such that this is not a reliable indicator of fate changes [30]. Finally, the distribution of Oct4^Δ/f^; Z/EG^+/−^; Bry-Cre^+/−^ cells did not appear altered. This suggests that any effect Oct4 has on cell fate either coincides with lineage specification or precedes it.

An alternative to an effect on cell fate specification is that Oct4 promotes expansion of unspecified progenitors by driving the cell cycle. Reduced mesenchyme density, decreased proliferation in the primitive streak, increased *Trp53* (p53) expression and increased *Cdkn1c* expression in the Oct4^COND MUT^ embryonic posterior all indicate that expansion of posterior progenitors is disrupted when Oct4 is depleted. The G1/S transition is effectively absent from ES cells, and binding of Oct4 to micro-RNAs that suppress inhibitors of the G1/S transition [68] may promote its bypass and limit the window for lineage-specific chromatin remodeling. Indeed, genes regulating ‘chromatin modification’ are up-regulated 24 hrs ATA coincident with reduced proliferation in the primitive streak (cluster 1–295: p = 2.1E-04 and cluster 613–908: p = 3.7E-04 using hypergeometric tests). Finally, c-Myc activates G1/S checkpoint complexes [69], [70], suggesting that c-Myc may be required to promote G1/S transition when the G1/S checkpoint is established coincident with Oct4 depletion.

Morrison and Brickman proposed that the evolutionarily conserved role of Oct4 might be facilitating expansion of progenitor populations during and after gastrulation based on work with paralogs: *Pou2* in *D. rerio* and *XlPou91* in *X. laevis* [64]. These *D. rerio Pou2* mutants [71] and *X. laevis* embryos treated with morpholinos against *XlPou91* share posterior truncations [64]. Since *Pou5f1* arose by duplication of *Pou2* [64], these data support a conserved role for Oct4 in posterior extension, which in mice includes maintaining proliferation in the primitive streak.

## Materials and Methods

### Animal husbandry

All procedures were approved by the University of Toronto Animal Care Committee in accordance with the Canadian Council on Animal Care. Foremost, both euthanasia and surgery were minimized. When performed, stress was minimized to the greatest extent possible before rapid depressive action on the CNS during euthanasia. Minimally invasive surgeries were performed under anesthetic to achieve complete depression of feedback from the PNS and analgesic used for recovery. For staging, embryos were assumed to be 0.5 days post coitum at 1pm on the day a vaginal plug was found. This is 12 hrs after the midpoint of the 14 hr light/10 hr dark cycle we used, where the lights were shut off every night at 8 pm and came on every morning at 6 am. Given the relevance of staging to this set of experiments, it is important to note that use of vaginal plugs –as opposed to direct observation of conception– is accompanied by ±7 hrs of variability in embryonic staging and is inferred from the midpoint of the dark period in the light/dark cycle. Embryos were dissected in Dulbecco's PBS (Gibco) and immediately placed in either liquid nitrogen (for microarrays and QPCR analysis) or in 4% paraformaldehyde (for sectioning and immunohistochemistry). Dissections for embryonic stages that are whole numbers (e.g. E8.0 or E9.0) were performed between 9 and 11 pm, while those occurring 12 hrs apart from whole days post coitum (e.g. E9.5 or E10.5) were performed between 12 and 2 pm. For the experiments assessing the timeframe of Oct4 depletion (Fig. 1D–G, S5A–D), tamoxifen was administered at 9 pm±30 min, and dissections performed the indicated number of hours ATA, e.g. dissections for the time-point 3 hrs ATA were done at midnight (12 am). The following stocks were used in the study: CD1 (Charles River), Oct4^f/f^ [25], lacZ/eGFP (Z/EG) [37], B6.Cg-Tg(Hist1H2BB/Egfp)1Pa/J (Histone H2B/eGFP fusion ‘HisGFP’) [38], Bry-Cre [36], Sox1-Cre [34], Foxa2^tm2.1(cre/Esr1*)Moon^/J [35], Sox2^f^ [39], CreER^T2^ [29]. Individual embryos or the associated extraembryonic tissues were genotyped as originally described.

Because a variety of experimental permutations were used in this project, the details of each permutation, including the mouse strains, genotypic ratios, tamoxifen administration regimen and other relevant features are provided on a separate row in Table S1 (the relevant row is noted as the experiment is described where ‘S1, row A’ is ‘S1A’).

### Tamoxifen administration

Tamoxifen was administered according to the protocol optimized following CreER^T2^ development [29]. 99 mg of tamoxifen (Sigma) was dissolved by sonication in a solution of 100 ul of ethanol (Sigma) and 1 ml of peanut seed oil (Sigma) [29]. The solution was kept in a ∼50°C water bath during preparation and prior to administration to avoid precipitation. 50 µl doses of this solution were administered to pregnant mothers by oral gavage using a 250 µl gastight #1725 syringe (Hamilton) [29]. Because of the uncertainty associated with staging embryos with vaginal plugs (±7 hrs), the time-point(s) indicated for tamoxifen administration are approximations, and listed as such (∼) within the text to reflect this uncertainty. In practice, tamoxifen was given at 9pm±30 min (∼E6.0, ∼E7.0 or ∼E8.0) or 9 am±30 min (∼E6.5, ∼E7.5 or ∼E8.5). The time-point(s) when tamoxifen was administered for each experimental permutation are listed in Table S1 as well as in the figure captions.

### Statistical analysis

The density of mesenchyme, frequency of apoptosis and proliferation, relative abundance of transcripts (other than Oct4), distance between neural folds and thickness of neuroepithelium were compared using 2-way ANOVAs. Depletion of Oct4 protein and transcript were compared with 1-way ANOVAs. F-values from the embryonic genotype's contribution (Oct4^f/f^ versus Oct4^f/f^;CreER^T2+/−^) to variation are indicated except for Figure 7K and S6C where the intra-embryo segment contribution is reported (e.g. difference between segments in the same embryo). Binding enrichment amongst differentially expressed genes and common causality of disrupted features in partially penetrant Oct4^COND MUT^ embryos was assessed using hypergeometric tests. The thickness of notochords was compared using a two-tailed t-test. A threshold of p<0.05 was used for each test (ANOVA, hypergeometric and t-test). Please see the Supplementary Methods (‘Text S1, page 1’) for detail on how measurements of Oct4 protein depletion, mesenchyme density, neuroepithelium thickness, notochord thickness, distance between the neural folds, and the fraction of Ph3+, Caspase-3+ and Oct4+ cells were taken (‘*Basic Measurements*’).

### Microscopy

Images in Figure 1F,G; Figure 2 F,G,I,J; Figure 3A–D; Fig 7A–J; Figure S6A,B,D,E; Figure S7A,B,D,E; Figure S8A,B and Figure S10A–F were taken with a Zeiss Axio Observer, images of Figure S5A–D were taken with an Olympus Fluoview 1000, images of Figure 2 B,C and Figure 4A–C were taken with an Olympus SZ61, and images of Figure 1A–C; Figure 3SA,B and Figure S4A were taken with a Leica MZ16 FA stereomicroscope. Contrast of the images in Figure 3D, 4A and 4C was enhanced with Adobe Photoshop v12.

### Immunohistochemistry and histology

Oct4 staining was performed as described previously [6]. For all other immunohistochemistry, embryos were fixed in 4% PFA overnight at 4°C, sectioned at a thickness of 10 µm and primary antibodies applied overnight at 4°C at the following concentrations: Oct-3/4 1∶200 (C-10 Santa Cruz), Chordin 1∶100 (R & D Systems), p-Smad1 1∶400 (Cell Signaling), Caspase-3 1∶500 (Promega), Ph3 1∶500 (Cell Signaling), Bry 1∶50 (R & D Systems), Sox2 1∶50 (R & D Systems). An antigen retrieval step of boiling the sample in 10 mM Sodium Citrate Buffer, pH 6.0 for 15 min was used for Oct-3/4 (C-10 immunofluorescent) and Chordin staining. Phalloidin staining (Alexa Fluor, Life Technologies) was performed according to the manufacturer's instructions. Hematoxylin and Eosin (Sigma) staining was performed according to the manufacturer's instructions.

### Quantitative PCR

Different litters from those used in the microarray analysis were used to confirm changes in gene expression by QPCR. Please see Supplementary Methods (‘Text S1, page 2) for assay details.

### Generating chimeras

Chimeras were produced as outlined in [72], and contribution was assessed by semi-quantitative PCR. Please see Supplementary Methods (‘Text S1,’ page 2) for details.

### Microarray and statistical enrichment analysis

RNA was extracted with Trizol according to the manufacturer's instructions (Invitrogen) and sent to the UHN Microarray Centre (Toronto, ON, Canada) for fluor-labeling (protocol GE2 v5.7), microarray hybridization, and array scanning. Please see Supplementary Methods (‘Text S1,’ page 4) for additional detail and analysis methodology.

### Basic measurements

Please see ‘Text S1.’

### Quantitative PCR

Please see ‘Text S1.’

### Generating chimeras

Please see ‘Text S1.’

### Measuring percent chimerism in diploid chimeras

Please see ‘Text S1.’

### Microarray and statistical enrichment analysis

Please see ‘Text S1.’

### Quantification of p-Smad1 Intensity

Please see ‘Text S1.’

## Supporting Information

Figure S1 Oct4 protein localization from E6.5–9.25 of murine development based on [6].
(TIF)

Figure S2 Oct4 transcript localization from E6.5–9.25 of murine development based on [7].
(TIF)

Figure S3 Tamoxifen administration ∼E7.5 and ∼E8.0 yields partial penetrance of the Oct4^COND MUT^ phenotype ∼E9.5 (Table S1B). Scale bars in ‘A,B’ are 1 mm. **A** Sagittal view of a representative Oct4^f/f^;CreER^T2+/−^ litter induced ∼E7.5 and dissected ∼E9.5. Penetrance of the Oct4^COND MUT^ phenotype is incomplete. The embryos are arranged such that phenotype severity declines in a clockwise fashion where ‘i’ has no phenotype, ‘ii’ has an open NT between closure points 1 and 2 that is marked with dashed line, and ‘iii–vii’ have the defects characteristic of Oct4^COND MUT^ embryos: truncated posteriors (compare ‘#’ ii versus iii), that has not turned (note how ‘ii’ faces its tail, whereas ‘iii–vii’ do not), and an open NT along its entire length marked in ‘iii’ with a dashed line. Neural tube closure of ‘iii’ is distinguished from ‘ii’ in that closure point 1 of embryo ‘ii’ is closed (indicated with a blue arrow), whereas this point fails to close in ‘iii.’ **B** Dorsal view of the same litter depicted in panel ‘A,’ without embryo ‘i.’ Neural tubes open along their entire length are evident in embryo ‘iii–vii’ (compare ‘ii’ to ‘iii–vii’). The distinguishing feature, closure at closure point 1 is highlighted: closure in ‘ii’ is indicated with an arrow, and failure to close in ‘iii’ is indicated with a pointed finger. **C** Breakdown of mutant features in each embryo induced ∼E7.5 and ∼E8.0.
(TIF)

Figure S4 Tamoxifen administration to Oct4^f/f^;CreER^T2+/−^ embryos ∼E6.0 yields a more severe phenotype than Oct4^COND MUT^ ∼E9.5 (Table S1C). **A,B** Phenotype after tamoxifen administration (ATA) ∼E6.0 and ∼E6.5 to Oct4^f/f^;CreER^T2+/−^ and dissection ∼E9.5. **A** WT Oct4^f/f^ E9.5 embryo for comparison. **B** Oct4^f/f^;CreER^T2+/^ embryos. The embryos are amorphous, where headfolds may be apparent (outlined in dashed blue line), but the remainder of the embryo does not develop.
(TIF)

Figure S5 Oct4 depletion is apparent by immunohistochemistry 20 hrs after tamoxifen administration (Table S1L). Scale bars in ‘A–D’ are 50 µm. **A–D** Comparison of the frequency of Oct4+ cells that are stained brown between Oct4^f/f^ and Oct4^f/f^;CreER^T2+/−^embryos. Nuclei are stained blue, anteriors are oriented to the left in each panel, and the region outlined with a red box in each panel ‘i’ is magnified and provided as an inset ‘ii’ in the upper right corner. **A,B** The frequency of Oct4+ cells is similar between Oct4^f/f^ and Oct4^f/f^;CreER^T2+/−^ embryos 15 hrs ATA. (A) Oct4^f/f^ (B) Oct4^f/f^;CreER^T2+/−^. **C,D** The frequency of Oct4+ cells declines 20 hrs ATA. (C) Oct4^f/f^ (D) Oct4^f/f^;CreER^T2+/−^.
(TIF)

Figure S6 A distal segment of neuroepithelium as well as notochords are thicker in E9.5 Oct4^COND MUT^ embryos (Table S1D). **A,B** Generally the region of thick neuroepithelium occurred in close proximity to where the first closure point would normally occur, dorsal to the first few somites. The red box in ‘B’ indicates a thick region of neuroepithelium in a Oct4^COND MUT^ embryo and an equivalent region in a control Oct4^f/f^ embryo is marked in ‘A.’ Both the embryos in ‘A’ and ‘B’ were induced with tamoxifen ∼E7.0 and ∼E7.5. The two-headed arrow indicates a sample measurement of neuroepithelial thickness. Embryos are oriented with the ventral side of each facing left. **C** Intra-embryo quantification of neuroepithelial thickness, comparing the distal region to adjacent regions. Error bars are ±s.e.m. Relative thickness (distal vs adjacent regions) within each embryo was compared with an ANOVA (F_1,287_ = 94.95, p<0.05 2-way ANOVA; ***p<0.001 Bonferroni posttest). **D–F** Cross-sections of Oct4^COND MUT^ notochords contain more cells. All litters ‘D–F’ were induced with tamoxifen ∼E7.0 and ∼E7.5 (Table S1D). Notochords are outlined with red boxes, and scale bars (D,E) are 50 µm. **D** Transverse section of notochord in an E9.5 Oct4^f/f^ embryo. **E** Transverse section of notochord in an Oct4^f/f^;CreER^T2+/−^ embryo. **F** Quantification of the average notochord thickness (cells/cross-section): Oct4^f/f^ vs Oct4^COND MUT^ (two-tailed t-test, *p<0.05).
(TIF)

Figure S7 Efficient Oct4 depletion during the sensitive window is required for penetrance of the Oct4^COND MUT^ phenotype. **A–C** Lineage specific recombinases induce lower rates of recombination than CreER^T2^ by the end of the developmental window in which embryos are sensitive to Oct4 loss (∼E7.5–E8.0) (Table S1R,S). Scale bars in ‘A,B’ are 50 µm. **A** Sagittal section of ∼E9.0 Sox1-Cre^+/−^;Z/EG^+/−^ embryo. **B** Sagittal section of ∼E9.0 Bry-Cre^+/−^;Z/EG^+/−^ embryo. Sections in ‘A’ and ‘B’ are oriented with the ventral side facing left. **C** Quantification of recombination frequency ∼E7.75 and ∼E9.0. **D,E** Localization of cells in which Bry-Cre induced recombination has occurred does not result in cellular diversion to neuroepithelium (Table S1U,V). **D** Sagittal sections of E9.5 embryos wherein one allele of Oct4 has been removed and the other is intact (Oct4^Δ/+^;Z/EG^+/−^;Bry-Cre^+/−^). GFP marks cells where recombination has occurred. **E** Sagittal section of E9.5 embryos wherein Oct4 has been depleted (Oct4^Δ/f^;Z/EG^+/−^;Bry-Cre^+/−^). The number of Bry^+^ cells are present in the neuroepithelium of E9.5 embryos is comparable to controls in ‘D’ which does not support diversion of cells into the neuroepithelium following Oct4 depletion. GFP marks cells where recombination has occurred. Magnified insets in the upper right correspond to the section outlined in each panel (D,E). Scale bars in ‘D’ and ‘E’ are 200 µm.
(TIF)

Figure S8 Specified lineages are present, appropriately localized, and proliferate at the same frequency as controls following Oct4 depletion (Table S1AC). The regions outlined with a white box in panel ‘i’ are magnified are magnified and provided as insets in panel ‘ii.’ Scale bars in ‘i’ are 50 µm, and are oriented such that the rostral end of the embryo is in the upper left of each panel ‘i’. The heart tube (*) and presumptive forebrain (%) are indicated to provide additional landmarks. **A** Brachyury is present in Oct4^f/f^;CreER^T2+/−^ embryos 48 hrs ATA, visible here in trunk mesenchyme (arrow). **B** Sox2 is present throughout the neuroepithelium (arrows) and gut (arrowhead) in the posterior of Oct4^f/f^;CreER^T2+/−^ embryos 48 hrs ATA. **C** Quantification of the fraction of lineage specified cells that are Ph3+ 48 hrs ATA (Oct4^f/f^;CreER^T2+/−^ vs Oct4^f/f^). Data are presented as mean ±s.e.m.
(TIF)

Figure S9 Sox2 transcript localization from E6.5–9.25 of murine development, based on [41].
(TIF)

Figure S10 The Chordin domain persists, while actin filament distribution appears altered by immunohistochemistry in Oct4^f/f^;CreER^T2+/−^ embryos following Oct4 depletion (Table S1AC). All litters depicted in panels ‘A–D’ were induced with tamoxifen ∼E7.0 and ∼E7.5. Scale bars in ‘A–D’ are 50 µm. **A,B** Specification of Chordin in the node occurs in spite of Oct4 depletion (36 hrs ATA). **A** Oct4^f/f^ **B** Oct4^f/f^;CreER^T2+/−^. **C,D** The distribution of actin appears altered 24 hrs ATA in Oct4^f/f^;CreER^T2+/−^ embryos. Arrows indicate region where anterior and posterior neuroepithelium of Oct4^f/f^;CreER^T2+/−^ embryos may adhere. **C** Oct4^f/f^ **D** Oct4^f/f^;CreER^T2+/−^.
(TIF)

Figure S11 Quantification of p-Smad1 intensity in Oct4^f/f^; CreER^T2+/−^ and Oct4^f/f^ embryos 36 hrs ATA suggests increased p-Smad1 following Oct4 depletion. Mean p-Smad1 intensity (multiple sections of the same embryo) in embryonic posteriors is depicted. The plot is aligned such that the intensity of p-Smad1 plotted on the x-axis shows p-Smad1 intensity (left to right; proximal to distal) in the embryonic posterior. The y-intersect, estimated based on morphology, is roughly where the allantois ends and the embryonic posterior begins.
(TIF)

Table S1 Each experimental permutation, including a description of the experiment, the mouse strains involved, appropriate references for these mouse strains, the parental cross, number of embryos/litters, genotypic ratios of progeny, phenotypes of progeny, tamoxifen administration regimen and other relevant features for each permutation are provided on a separate row (the relevant row is noted in the manuscript as the experiment is described, where ‘S1, row A’ is noted as ‘S1A’ in the main text).
(XLS)

Table S2 Differential expression (24, 36, or 48 hrs ATA) amongst genes that physically interact with Oct4 in ES cells following Oct4 depletion ∼E7.5.
(XLSX)

Text S1 Supplementary Methods. The supplementary methods section provides additional methodological detail related to: measurements made in the manuscript (timecourse of Oct4 protein depletion, mesenchyme density, neuroepithelial thickness, notochord thickness, distance between neural folds as well as Ph3+ and Caspase-3+ populations), Quantitative PCR (assay details), a summary of how chimeras were generated and the chimeric contribution quantified, detail concerning how the samples were prepared for microarrays and how the output of these assays was statistically analyzed. Supplementary references related to this methodology are also included.
(DOC)

## References

[1] Nichols J, Zevnik B, Anastassiadis K, Niwa H, Klewe-Nebenius D, et al (1998) Formation of pluripotent stem cells in the mammalian embryo depends on the POU transcription factor Oct4. Cell 95: 379–391. 9814708 10.1016/s0092-8674(00)81769-9

[2] Wang J, Rao S, Chu J, Shen X, Levasseur DN, et al (2006) A protein interaction network for pluripotency of embryonic stem cells. Nature 444: 364–368. 17093407 10.1038/nature05284

[3] Liang J, Wan M, Zhang Y, Gu P, Xin H, et al (2008) Nanog and Oct4 associate with unique transcriptional repression complexes in embryonic stem cells. Nat Cell Biol 10: 731–739. 18454139 10.1038/ncb1736

[4] Pardo M, Lang B, Yu L, Prosser H, Bradley A, et al (2010) An expanded Oct4 interaction network: implications for stem cell biology, development, and disease. Cell Stem Cell 6: 382–395. 20362542 10.1016/j.stem.2010.03.004 PMC2860244

[5] Chen X, Xu H, Yuan P, Fang F, Huss M, et al (2008) Integration of external signaling pathways with the core transcriptional network in embryonic stem cells. Cell 133: 1106–1117. 18555785 10.1016/j.cell.2008.04.043

[6] Downs KM (2008) Systematic localization of Oct-3/4 to the gastrulating mouse conceptus suggests manifold roles in mammalian development. Dev Dyn 237: 464–475. 18213575 10.1002/dvdy.21438

[7] Scholer HR, Dressler GR, Balling R, Rohdewohld H, Gruss P (1990) Oct-4: a germline-specific transcription factor mapping to the mouse t-complex. EMBO J 9: 2185–2195. 2357966 10.1002/j.1460-2075.1990.tb07388.x PMC551941

[8] Pesce M, Wang X, Wolgemuth DJ, Scholer H (1998) Differential expression of the Oct-4 transcription factor during mouse germ cell differentiation. Mech Dev 71: 89–98. 9507072 10.1016/s0925-4773(98)00002-1

[9] Yoshimizu T, Sugiyama N, De Felice M, Yeom YI, Ohbo K, et al (1999) Germline-specific expression of the Oct-4/green fluorescent protein (GFP) transgene in mice. Dev Growth Differ 41: 675–684. 10646797 10.1046/j.1440-169x.1999.00474.x

[10] Bullejos M, Koopman P (2004) Germ cells enter meiosis in a rostro-caudal wave during development of the mouse ovary. Mol Reprod Dev 68: 422–428. 15236325 10.1002/mrd.20105

[11] Brennan J, Lu CC, Norris DP, Rodriguez TA, Beddington RS, et al (2001) Nodal signalling in the epiblast patterns the early mouse embryo. Nature 411: 965–969. 11418863 10.1038/35082103

[12] Waldrip WR, Bikoff EK, Hoodless PA, Wrana JL, Robertson EJ (1998) Smad2 signaling in extraembryonic tissues determines anterior-posterior polarity of the early mouse embryo. Cell 92: 797–808. 9529255 10.1016/s0092-8674(00)81407-5

[13] Fuhrmann G, Chung AC, Jackson KJ, Hummelke G, Baniahmad A, et al (2001) Mouse germline restriction of Oct4 expression by germ cell nuclear factor. Dev Cell 1: 377–387. 11702949 10.1016/s1534-5807(01)00038-7

[14] Teo AK, Arnold SJ, Trotter MW, Brown S, Ang LT, et al (2011) Pluripotency factors regulate definitive endoderm specification through eomesodermin. Genes Dev 25: 238–250. 21245162 10.1101/gad.607311 PMC3034899

[15] Niwa H, Sekita Y, Tsend-Ayush E, Grutzner F (2008) Platypus Pou5f1 reveals the first steps in the evolution of trophectoderm differentiation and pluripotency in mammals. Evol Dev 10: 671–682. 19021737 10.1111/j.1525-142X.2008.00280.x

[16] Frankenberg S, Pask A, Renfree MB (2010) The evolution of class V POU domain transcription factors in vertebrates and their characterisation in a marsupial. Dev Biol 337: 162–170. 19850032 10.1016/j.ydbio.2009.10.017

[17] Beddington RS (1983) Histogenetic and neoplastic potential of different regions of the mouse embryonic egg cylinder. J Embryol Exp Morphol 75: 189–204. 6886610

[18] Damjanov I, Solter D, Skreb N (1971) Teratocarcinogenesis as related to the age of embryos grafted under the kidney capsule. Wilhelm Roux' Archiv für Entwicklungsmechanik der Organismen 167: 288–290. 10.1007/BF00584254 28304666

[19] Osorno R, Tsakiridis A, Wong F, Cambray N, Economou C, et al The developmental dismantling of pluripotency is reversed by ectopic Oct4 expression. Development 139: 2288–2298. 22669820 10.1242/dev.078071 PMC3367440

[20] Thomson M, Liu SJ, Zou LN, Smith Z, Meissner A, et al (2011) Pluripotency factors in embryonic stem cells regulate differentiation into germ layers. Cell 145: 875–889. 21663792 10.1016/j.cell.2011.05.017 PMC5603300

[21] Niwa H, Miyazaki J, Smith AG (2000) Quantitative expression of Oct-3/4 defines differentiation, dedifferentiation or self-renewal of ES cells. Nat Genet 24: 372–376. 10742100 10.1038/74199

[22] Zeineddine D, Papadimou E, Chebli K, Gineste M, Liu J, et al (2006) Oct-3/4 dose dependently regulates specification of embryonic stem cells toward a cardiac lineage and early heart development. Dev Cell 11: 535–546. 17011492 10.1016/j.devcel.2006.07.013

[23] Shimozaki K, Nakashima K, Niwa H, Taga T (2003) Involvement of Oct3/4 in the enhancement of neuronal differentiation of ES cells in neurogenesis-inducing cultures. Development 130: 2505–2512. 12702663 10.1242/dev.00476

[24] Yuan H, Corbi N, Basilico C, Dailey L (1995) Developmental-specific activity of the FGF-4 enhancer requires the synergistic action of Sox2 and Oct-3. Genes Dev 9: 2635–2645. 7590241 10.1101/gad.9.21.2635

[25] Kehler J, Tolkunova E, Koschorz B, Pesce M, Gentile L, et al (2004) Oct4 is required for primordial germ cell survival. EMBO Rep 5: 1078–1083. 15486564 10.1038/sj.embor.7400279 PMC1299174

[26] Ho L, Ronan JL, Wu J, Staahl BT, Chen L, et al (2009) An embryonic stem cell chromatin remodeling complex, esBAF, is essential for embryonic stem cell self-renewal and pluripotency. Proc Natl Acad Sci U S A 106: 5181–5186. 19279220 10.1073/pnas.0812889106 PMC2654396

[27] van den Berg DL, Snoek T, Mullin NP, Yates A, Bezstarosti K, et al (2010) An Oct4-centered protein interaction network in embryonic stem cells. Cell Stem Cell 6: 369–381. 20362541 10.1016/j.stem.2010.02.014 PMC2860243

[28] Pesce M, Scholer HR (2001) Oct-4: gatekeeper in the beginnings of mammalian development. Stem Cells 19: 271–278. 11463946 10.1634/stemcells.19-4-271

[29] Seibler J, Zevnik B, Kuter-Luks B, Andreas S, Kern H, et al (2003) Rapid generation of inducible mouse mutants. Nucleic Acids Res 31: e12. 12582257 10.1093/nar/gng012 PMC150244

[30] Copp AJ, Greene ND, Murdoch JN (2003) The genetic basis of mammalian neurulation. Nat Rev Genet 4: 784–793. 13679871 10.1038/nrg1181

[31] Kalantry S, Manning S, Haub O, Tomihara-Newberger C, Lee HG, et al (2001) The amnionless gene, essential for mouse gastrulation, encodes a visceral-endoderm-specific protein with an extracellular cysteine-rich domain. Nat Genet 27: 412–416. 11279523 10.1038/86912

[32] Nagy A, Gocza E, Diaz EM, Prideaux VR, Ivanyi E, et al (1990) Embryonic stem cells alone are able to support fetal development in the mouse. Development 110: 815–821. 2088722 10.1242/dev.110.3.815

[33] McLaren A, Michie D (1956) Studies on the transfer of fertilized mouse eggs to the uterine foster-mothers I. Factors affecting the implantation and survival of native and transferred eggs. The Journal of Experimental Biology 33: 394–416.

[34] Takashima Y, Era T, Nakao K, Kondo S, Kasuga M, et al (2007) Neuroepithelial cells supply an initial transient wave of MSC differentiation. Cell 129: 1377–1388. 17604725 10.1016/j.cell.2007.04.028

[35] Park EJ, Sun X, Nichol P, Saijoh Y, Martin JF, et al (2008) System for tamoxifen-inducible expression of cre-recombinase from the Foxa2 locus in mice. Dev Dyn 237: 447–453. 18161057 10.1002/dvdy.21415

[36] Feller J, Schneider A, Schuster-Gossler K, Gossler A (2008) Noncyclic Notch activity in the presomitic mesoderm demonstrates uncoupling of somite compartmentalization and boundary formation. Genes Dev 22: 2166–2171. 18708576 10.1101/gad.480408 PMC2518812

[37] Novak A, Guo C, Yang W, Nagy A, Lobe CG (2000) Z/EG, a double reporter mouse line that expresses enhanced green fluorescent protein upon Cre-mediated excision. Genesis 28: 147–155. 11105057

[38] Hadjantonakis AK, Papaioannou VE (2004) Dynamic in vivo imaging and cell tracking using a histone fluorescent protein fusion in mice. BMC Biotechnol 4: 33. 15619330 10.1186/1472-6750-4-33 PMC544401

[39] Taranova OV, Magness ST, Fagan BM, Wu Y, Surzenko N, et al (2006) SOX2 is a dose-dependent regulator of retinal neural progenitor competence. Genes Dev 20: 1187–1202. 16651659 10.1101/gad.1407906 PMC1472477

[40] Masui S, Nakatake Y, Toyooka Y, Shimosato D, Yagi R, et al (2007) Pluripotency governed by Sox2 via regulation of Oct3/4 expression in mouse embryonic stem cells. Nat Cell Biol 9: 625–635. 17515932 10.1038/ncb1589

[41] Avilion AA, Nicolis SK, Pevny LH, Perez L, Vivian N, et al (2003) Multipotent cell lineages in early mouse development depend on SOX2 function. Genes Dev 17: 126–140. 12514105 10.1101/gad.224503 PMC195970

[42] Kim J, Chu J, Shen X, Wang J, Orkin SH (2008) An extended transcriptional network for pluripotency of embryonic stem cells. Cell 132: 1049–1061. 18358816 10.1016/j.cell.2008.02.039 PMC3837340

[43] Marson A, Levine SS, Cole MF, Frampton GM, Brambrink T, et al (2008) Connecting microRNA genes to the core transcriptional regulatory circuitry of embryonic stem cells. Cell 134: 521–533. 18692474 10.1016/j.cell.2008.07.020 PMC2586071

[44] Kiermer V (2006) Embryos and biopsies on the ChIP-ing forecast. Nat Methods 3: 583. 16892524 10.1038/nmeth0806-583

[45] Nakatake Y, Fukui N, Iwamatsu Y, Masui S, Takahashi K, et al (2006) Klf4 cooperates with Oct3/4 and Sox2 to activate the Lefty1 core promoter in embryonic stem cells. Mol Cell Biol 26: 7772–7782. 16954384 10.1128/MCB.00468-06 PMC1636862

[46] Hall J, Guo G, Wray J, Eyres I, Nichols J, et al (2009) Oct4 and LIF/Stat3 additively induce Kruppel factors to sustain embryonic stem cell self-renewal. Cell Stem Cell 5: 597–609. 19951688 10.1016/j.stem.2009.11.003

[47] Yi F, Pereira L, Hoffman JA, Shy BR, Yuen CM, et al (2011) Opposing effects of Tcf3 and Tcf1 control Wnt stimulation of embryonic stem cell self-renewal. Nat Cell Biol 13: 762–770. 21685894 10.1038/ncb2283 PMC3129424

[48] Sylvester I, Scholer HR (1994) Regulation of the Oct-4 gene by nuclear receptors. Nucleic Acids Res 22: 901–911. 8152920 10.1093/nar/22.6.901 PMC307908

[49] Jung JS, Jee MK, Cho HT, Choi JI, Im YB, et al MBD6 is a direct target of Oct4 and controls the stemness and differentiation of adipose tissue-derived stem cells. Cell Mol Life Sci 70: 711–728. 23052207 10.1007/s00018-012-1157-4 PMC11114067

[50] Massague J, Seoane J, Wotton D (2005) Smad transcription factors. Genes Dev 19: 2783–2810. 16322555 10.1101/gad.1350705

[51] Beddington RS (1994) Induction of a second neural axis by the mouse node. Development 120: 613–620. 8162859 10.1242/dev.120.3.613

[52] Przemeck GK, Heinzmann U, Beckers J, Hrabe de Angelis M (2003) Node and midline defects are associated with left-right development in Delta1 mutant embryos. Development 130: 3–13. 12441287 10.1242/dev.00176

[53] Tam PP, Behringer RR (1997) Mouse gastrulation: the formation of a mammalian body plan. Mech Dev 68: 3–25. 9431800 10.1016/s0925-4773(97)00123-8

[54] Conlon FL, Lyons KM, Takaesu N, Barth KS, Kispert A, et al (1994) A primary requirement for nodal in the formation and maintenance of the primitive streak in the mouse. Development 120: 1919–1928. 7924997 10.1242/dev.120.7.1919

[55] Chu GC, Dunn NR, Anderson DC, Oxburgh L, Robertson EJ (2004) Differential requirements for Smad4 in TGFbeta-dependent patterning of the early mouse embryo. Development 131: 3501–3512. 15215210 10.1242/dev.01248

[56] Tremblay KD, Hoodless PA, Bikoff EK, Robertson EJ (2000) Formation of the definitive endoderm in mouse is a Smad2-dependent process. Development 127: 3079–3090. 10862745 10.1242/dev.127.14.3079

[57] Zhou X, Sasaki H, Lowe L, Hogan BL, Kuehn MR (1993) Nodal is a novel TGF-beta-like gene expressed in the mouse node during gastrulation. Nature 361: 543–547. 8429908 10.1038/361543a0

[58] Furtado MB, Solloway MJ, Jones VJ, Costa MW, Biben C, et al (2008) BMP/SMAD1 signaling sets a threshold for the left/right pathway in lateral plate mesoderm and limits availability of SMAD4. Genes Dev 22: 3037–3049. 18981480 10.1101/gad.1682108 PMC2577791

[59] Collignon J, Varlet I, Robertson EJ (1996) Relationship between asymmetric nodal expression and the direction of embryonic turning. Nature 381: 155–158. 8610012 10.1038/381155a0

[60] Candia AF, Watabe T, Hawley SH, Onichtchouk D, Zhang Y, et al (1997) Cellular interpretation of multiple TGF-beta signals: intracellular antagonism between activin/BVg1 and BMP-2/4 signaling mediated by Smads. Development 124: 4467–4480. 9409665 10.1242/dev.124.22.4467

[61] Tzouanacou E, Wegener A, Wymeersch FJ, Wilson V, Nicolas JF (2009) Redefining the progression of lineage segregations during mammalian embryogenesis by clonal analysis. Dev Cell 17: 365–376. 19758561 10.1016/j.devcel.2009.08.002

[62] Miyagi S, Masui S, Niwa H, Saito T, Shimazaki T, et al (2008) Consequence of the loss of Sox2 in the developing brain of the mouse. FEBS Lett 582: 2811–2815. 18638478 10.1016/j.febslet.2008.07.011

[63] Schoenwolf GC, Desmond ME (1984) Neural tube occlusion precedes rapid brain enlargement. J Exp Zool 230: 405–407. 6747570 10.1002/jez.1402300309

[64] Morrison GM, Brickman JM (2006) Conserved roles for Oct4 homologues in maintaining multipotency during early vertebrate development. Development 133: 2011–2022. 16651543 10.1242/dev.02362

[65] Snir M, Ofir R, Elias S, Frank D (2006) Xenopus laevis POU91 protein, an Oct3/4 homologue, regulates competence transitions from mesoderm to neural cell fates. EMBO J 25: 3664–3674. 16858397 10.1038/sj.emboj.7601238 PMC1538554

[66] Chapman DL, Papaioannou VE (1998) Three neural tubes in mouse embryos with mutations in the T-box gene Tbx6. Nature 391: 695–697. 9490412 10.1038/35624

[67] Yoshikawa Y, Fujimori T, McMahon AP, Takada S (1997) Evidence that absence of Wnt-3a signaling promotes neuralization instead of paraxial mesoderm development in the mouse. Dev Biol 183: 234–242. 9126297 10.1006/dbio.1997.8502

[68] Wang Y, Blelloch R (2009) Cell cycle regulation by MicroRNAs in embryonic stem cells. Cancer Res 69: 4093–4096. 19435891 10.1158/0008-5472.CAN-09-0309 PMC2894693

[69] Kim J, Woo AJ, Chu J, Snow JW, Fujiwara Y, et al (2010) A Myc network accounts for similarities between embryonic stem and cancer cell transcription programs. Cell 143: 313–324. 20946988 10.1016/j.cell.2010.09.010 PMC3018841

[70] Mateyak MK, Obaya AJ, Sedivy JM (1999) c-Myc regulates cyclin D-Cdk4 and -Cdk6 activity but affects cell cycle progression at multiple independent points. Mol Cell Biol 19: 4672–4683. 10373516 10.1128/mcb.19.7.4672 PMC84265

[71] Reim G, Mizoguchi T, Stainier DY, Kikuchi Y, Brand M (2004) The POU domain protein spg (pou2/Oct4) is essential for endoderm formation in cooperation with the HMG domain protein casanova. Dev Cell 6: 91–101. 14723850 10.1016/s1534-5807(03)00396-4

[72] Wood SA, Allen ND, Rossant J, Auerbach A, Nagy A (1993) Non-injection methods for the production of embryonic stem cell-embryo chimaeras. Nature 365: 87–89. 8361547 10.1038/365087a0

