# Supplementary figures and images for: Oct4 Is Required ∼E7.5 for Proliferation in the Primitive Streak
Source: PLoS Genet. 2013 Nov 14;9(11):e1003957. doi: 10.1371/journal.pgen.1003957 (PMC3828132; doi:10.1371/journal.pgen.1003957)

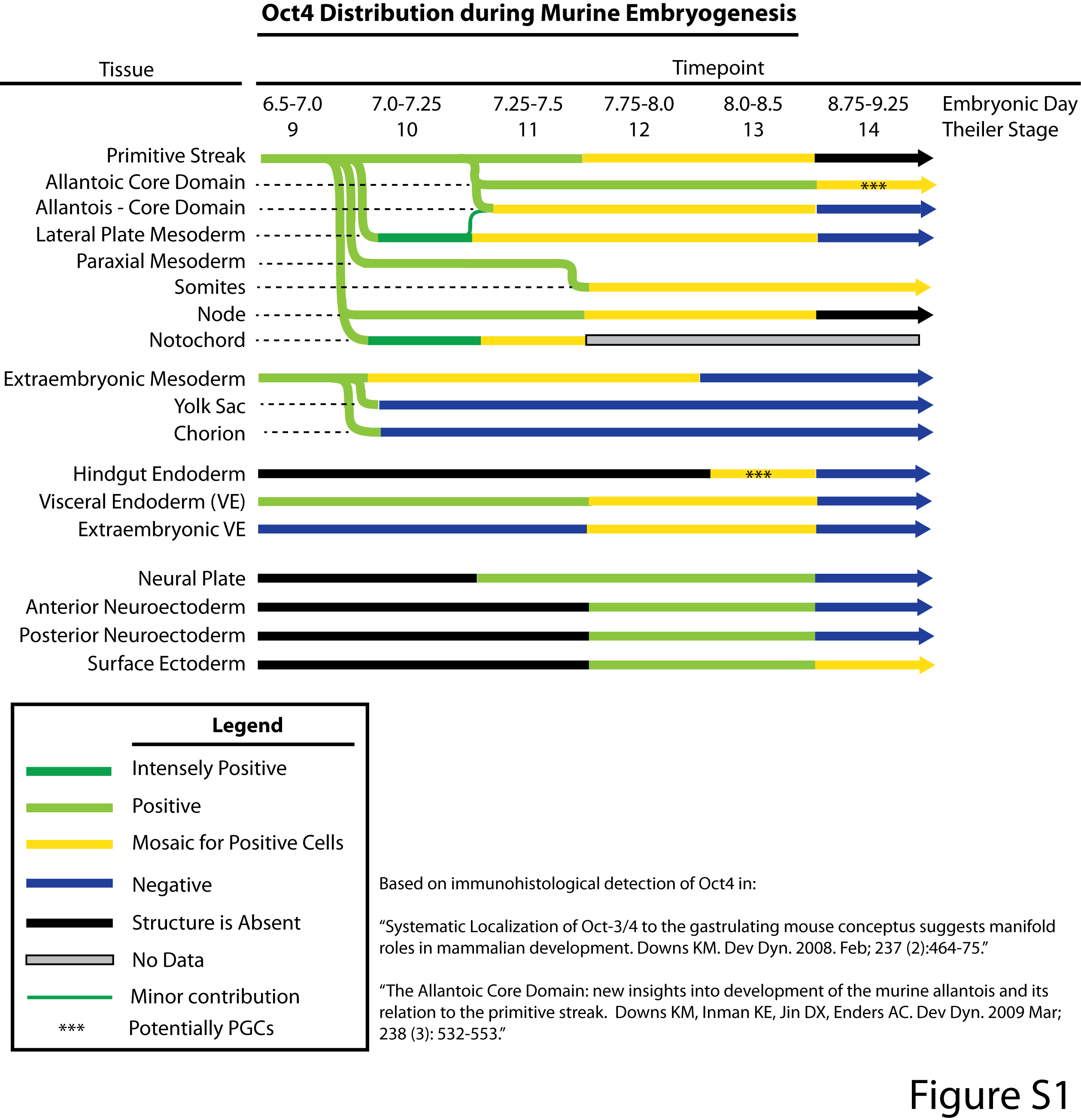

Supplement: Figure S1 — Oct4 protein localization from E6.5–9.25 of murine development based on [6]. (TIF) [file pgen.1003957.s001.tif]

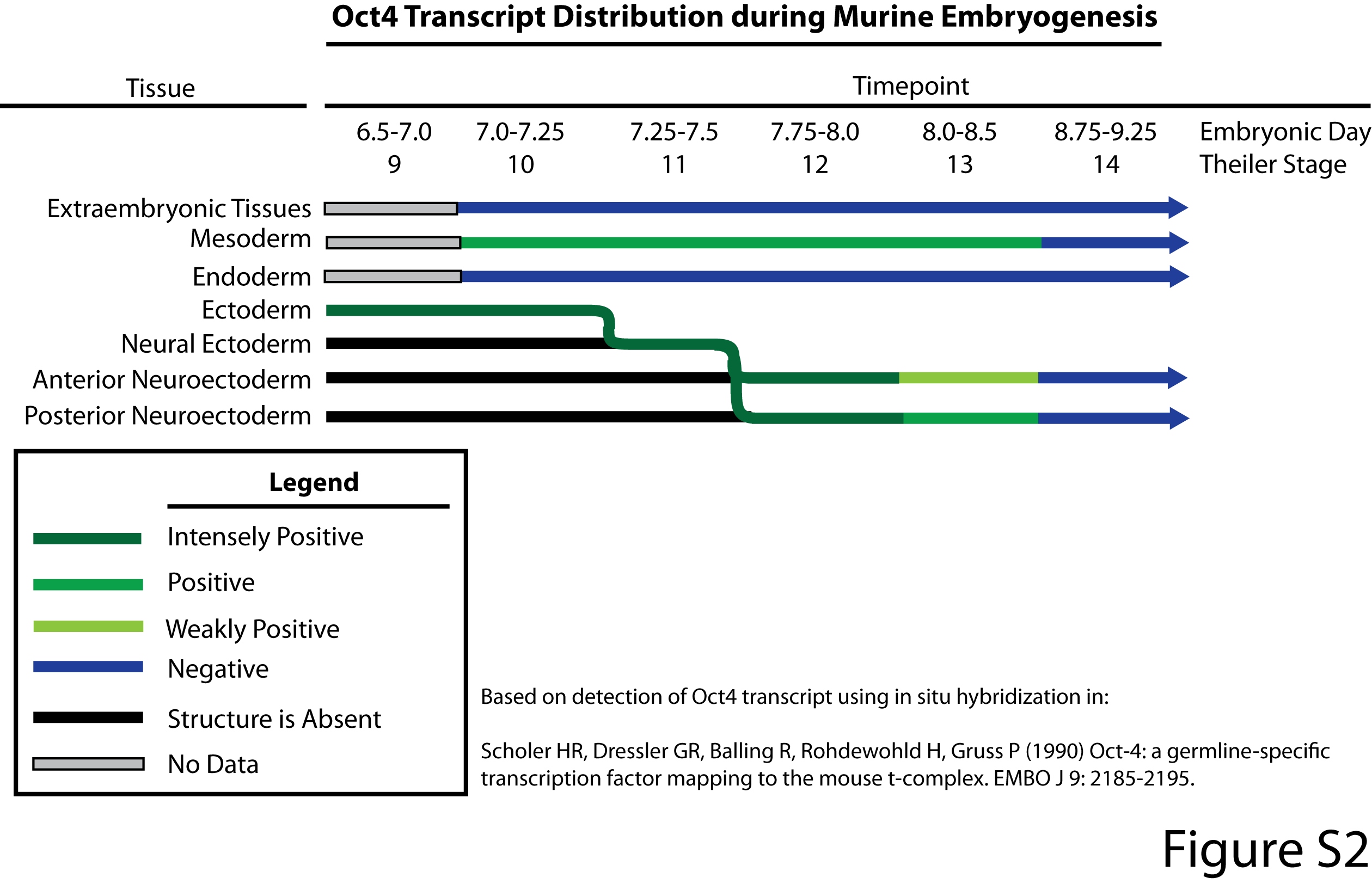

Supplement: Figure S2 — Oct4 transcript localization from E6.5–9.25 of murine development based on [7]. (TIF) [file pgen.1003957.s002.tif]

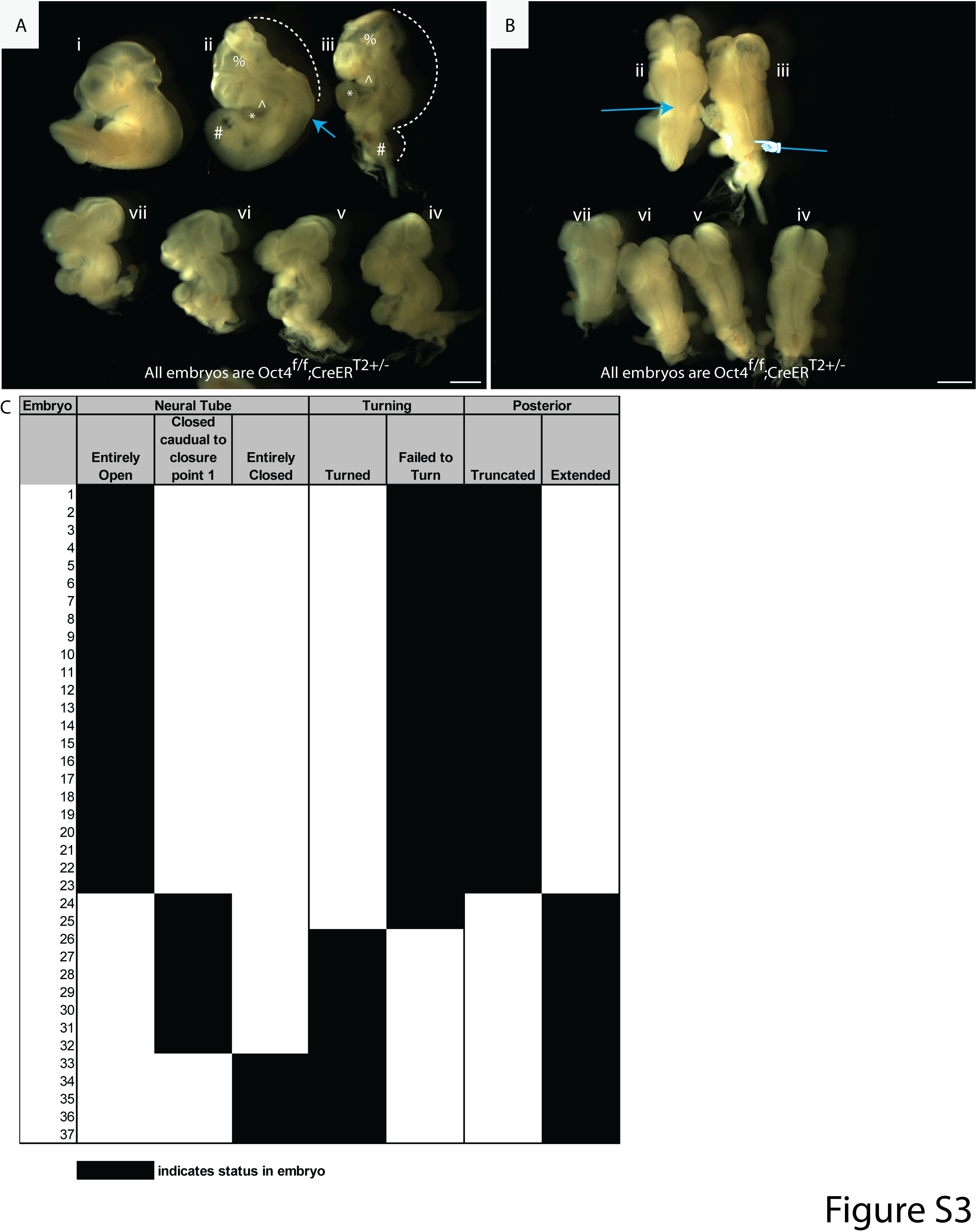

Supplement: Figure S3 — Tamoxifen administration ∼E7.5 and ∼E8.0 yields partial penetrance of the Oct4COND MUT phenotype ∼E9.5 (Table S1B). Scale bars in ‘A,B’ are 1 mm. A Sagittal view of a representative Oct4f/f;CreERT2+/− litter induced ∼E7.5 and dissected ∼E9.5. Penetrance of the Oct4COND MUT phenotype is incomplete. The embryos are arranged such that phenotype severity declines in a clockwise fashion where ‘i’ has no phenotype, ‘ii’ has an open NT between closure points 1 and 2 that is marked with dashed line, and ‘iii–vii’ have the defects characteristic of Oct4COND MUT embryos: truncated posteriors (compare ‘#’ ii versus iii), that has not turned (note how ‘ii’ faces its tail, whereas ‘iii–vii’ do not), and an open NT along its entire length marked in ‘iii’ with a dashed line. Neural tube closure of ‘iii’ is distinguished from ‘ii’ in that closure point 1 of embryo ‘ii’ is closed (indicated with a blue arrow), whereas this point fails to close in ‘iii.’ B Dorsal view of the same litter depicted in panel ‘A,’ without embryo ‘i.’ Neural tubes open along their entire length are evident in embryo ‘iii–vii’ (compare ‘ii’ to ‘iii–vii’). The distinguishing feature, closure at closure point 1 is highlighted: closure in ‘ii’ is indicated with an arrow, and failure to close in ‘iii’ is indicated with a pointed finger. C Breakdown of mutant features in each embryo induced ∼E7.5 and ∼E8.0. (TIF) [file pgen.1003957.s003.tif]

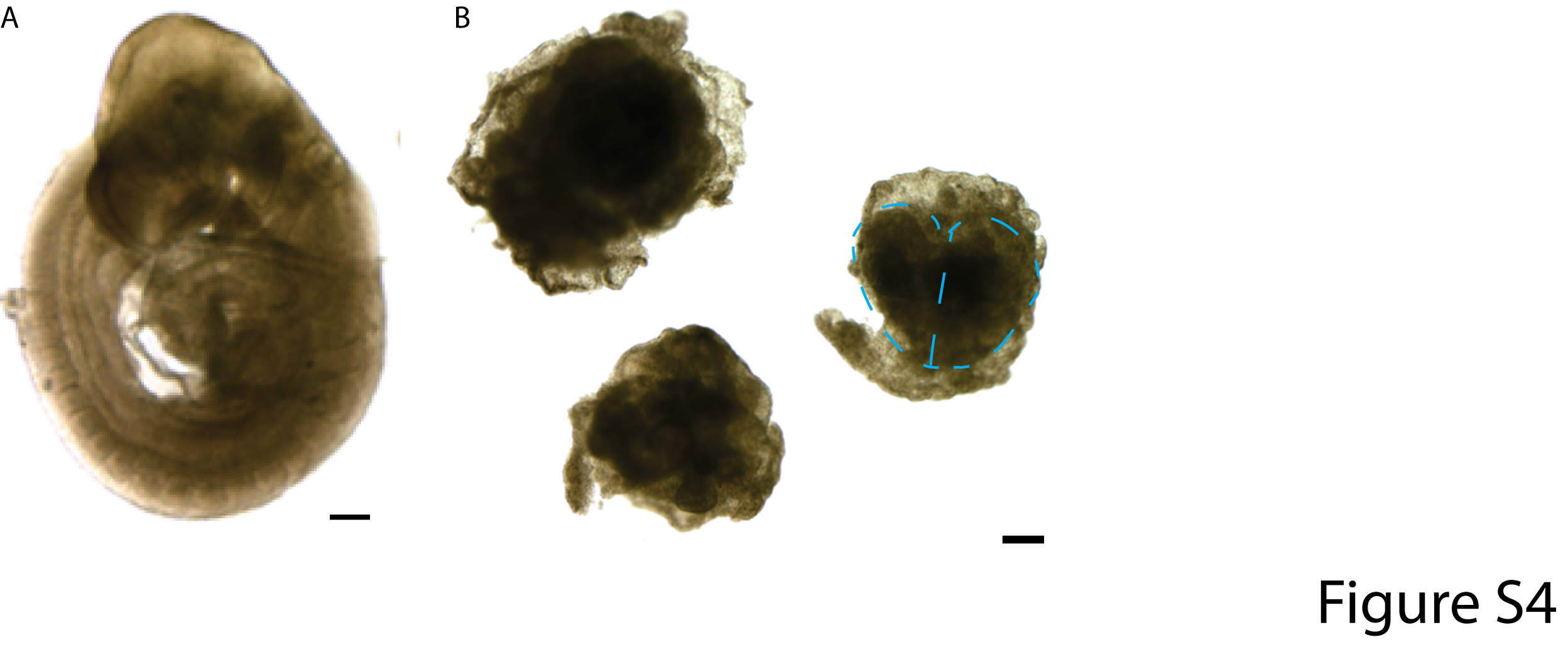

Supplement: Figure S4 — Tamoxifen administration to Oct4f/f;CreERT2+/− embryos ∼E6.0 yields a more severe phenotype than Oct4COND MUT ∼E9.5 (Table S1C). A,B Phenotype after tamoxifen administration (ATA) ∼E6.0 and ∼E6.5 to Oct4f/f;CreERT2+/− and dissection ∼E9.5. A WT Oct4f/f E9.5 embryo for comparison. B Oct4f/f;CreERT2+/ embryos. The embryos are amorphous, where headfolds may be apparent (outlined in dashed blue line), but the remainder of the embryo does not develop. (TIF) [file pgen.1003957.s004.tif]

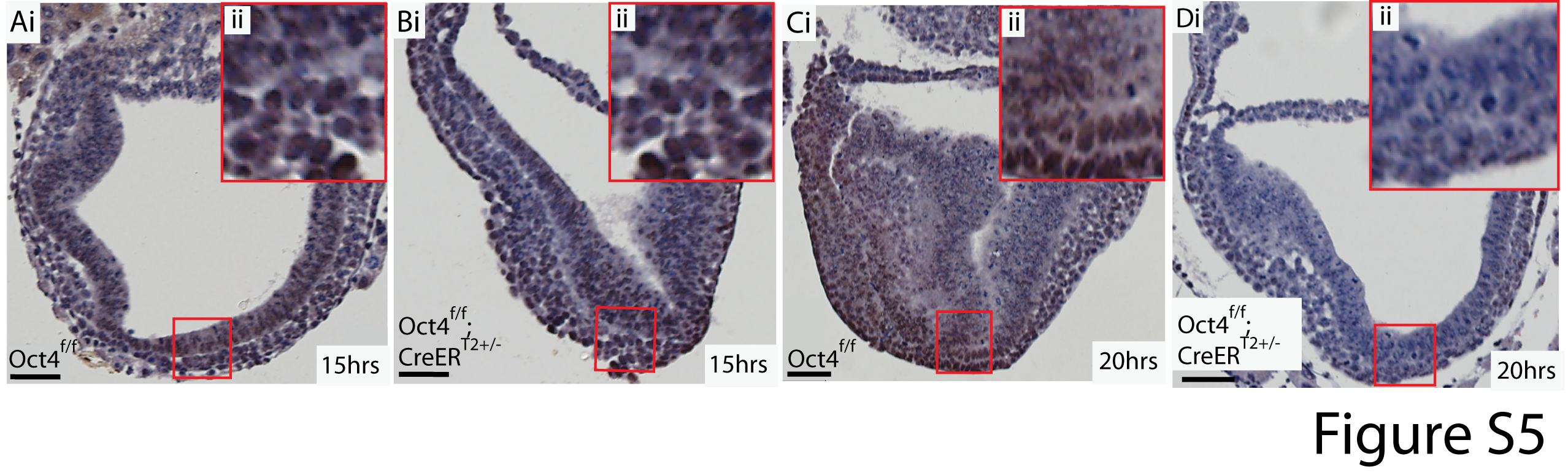

Supplement: Figure S5 — Oct4 depletion is apparent by immunohistochemistry 20 hrs after tamoxifen administration (Table S1L). Scale bars in ‘A–D’ are 50 µm. A–D Comparison of the frequency of Oct4+ cells that are stained brown between Oct4f/f and Oct4f/f;CreERT2+/−embryos. Nuclei are stained blue, anteriors are oriented to the left in each panel, and the region outlined with a red box in each panel ‘i’ is magnified and provided as an inset ‘ii’ in the upper right corner. A,B The frequency of Oct4+ cells is similar between Oct4f/f and Oct4f/f;CreERT2+/− embryos 15 hrs ATA. (A) Oct4f/f (B) Oct4f/f;CreERT2+/−. C,D The frequency of Oct4+ cells declines 20 hrs ATA. (C) Oct4f/f (D) Oct4f/f;CreERT2+/−. (TIF) [file pgen.1003957.s005.tif]

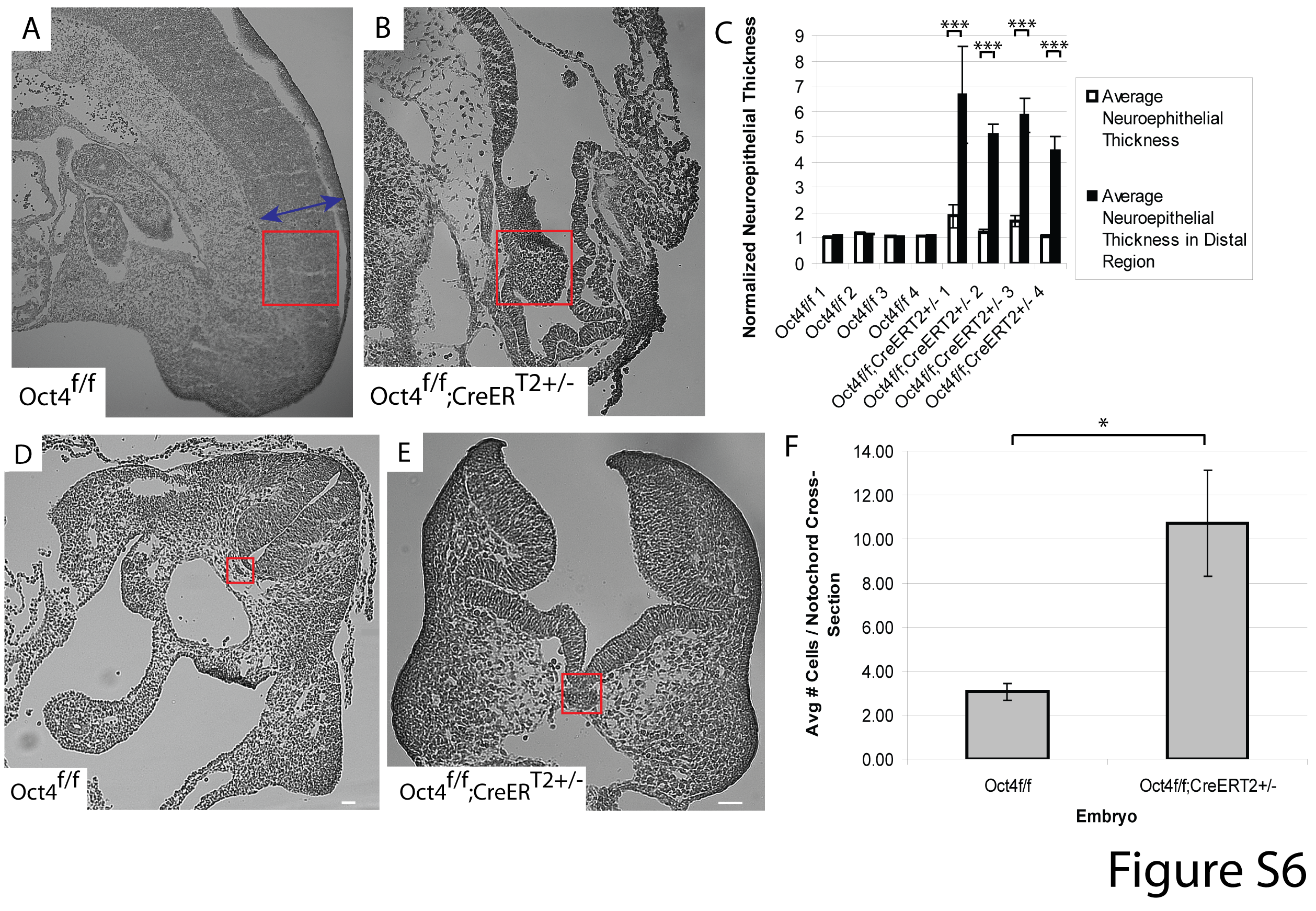

Supplement: Figure S6 — A distal segment of neuroepithelium as well as notochords are thicker in E9.5 Oct4COND MUT embryos (Table S1D). A,B Generally the region of thick neuroepithelium occurred in close proximity to where the first closure point would normally occur, dorsal to the first few somites. The red box in ‘B’ indicates a thick region of neuroepithelium in a Oct4COND MUT embryo and an equivalent region in a control Oct4f/f embryo is marked in ‘A.’ Both the embryos in ‘A’ and ‘B’ were induced with tamoxifen ∼E7.0 and ∼E7.5. The two-headed arrow indicates a sample measurement of neuroepithelial thickness. Embryos are oriented with the ventral side of each facing left. C Intra-embryo quantification of neuroepithelial thickness, comparing the distal region to adjacent regions. Error bars are ±s.e.m. Relative thickness (distal vs adjacent regions) within each embryo was compared with an ANOVA (F1,287 = 94.95, p<0.05 2-way ANOVA; ***p<0.001 Bonferroni posttest). D–F Cross-sections of Oct4COND MUT notochords contain more cells. All litters ‘D–F’ were induced with tamoxifen ∼E7.0 and ∼E7.5 (Table S1D). Notochords are outlined with red boxes, and scale bars (D,E) are 50 µm. D Transverse section of notochord in an E9.5 Oct4f/f embryo. E Transverse section of notochord in an Oct4f/f;CreERT2+/− embryo. F Quantification of the average notochord thickness (cells/cross-section): Oct4f/f vs Oct4COND MUT (two-tailed t-test, *p<0.05). (TIF) [file pgen.1003957.s006.tif]

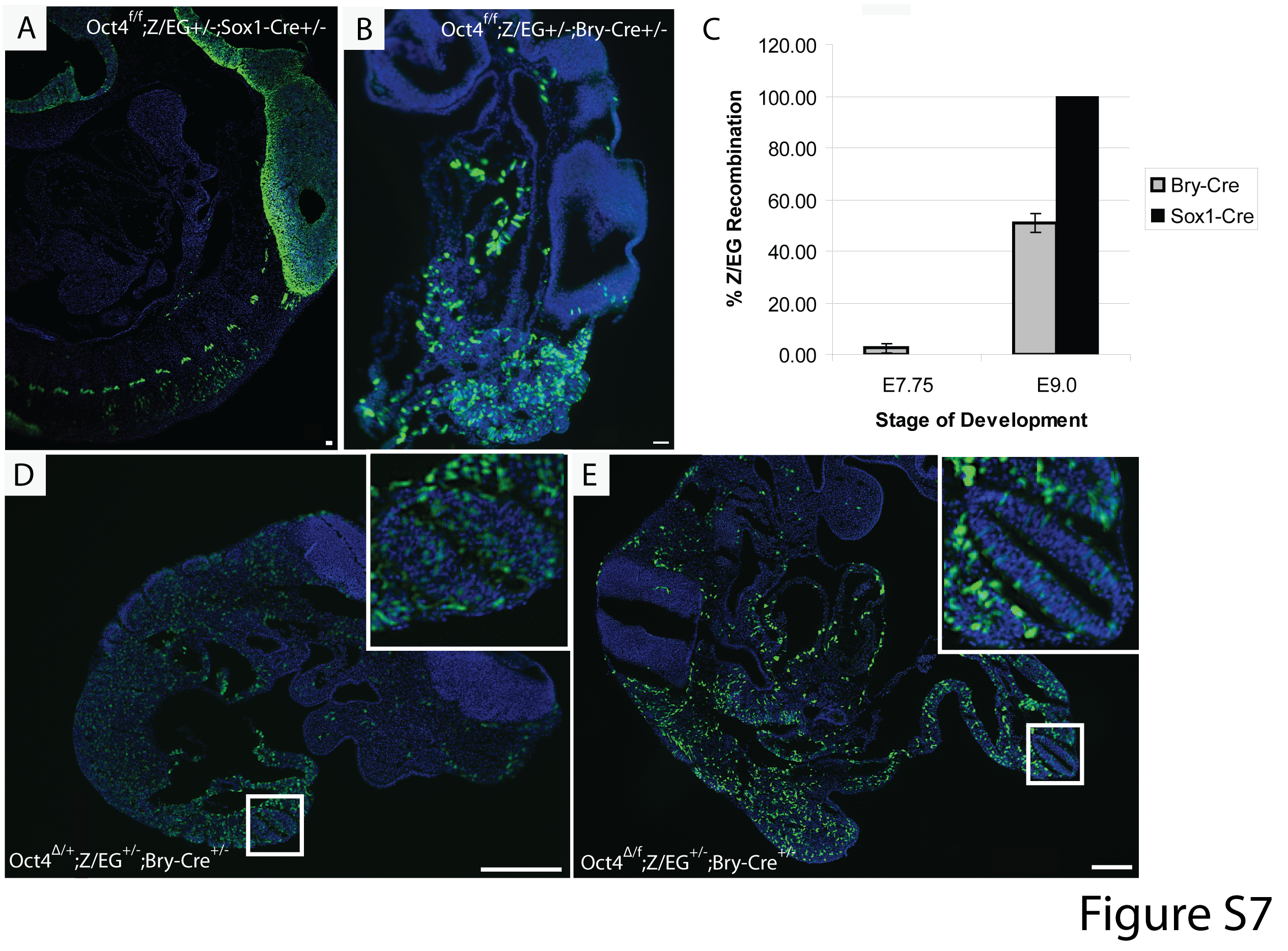

Supplement: Figure S7 — Efficient Oct4 depletion during the sensitive window is required for penetrance of the Oct4COND MUT phenotype. A–C Lineage specific recombinases induce lower rates of recombination than CreERT2 by the end of the developmental window in which embryos are sensitive to Oct4 loss (∼E7.5–E8.0) (Table S1R,S). Scale bars in ‘A,B’ are 50 µm. A Sagittal section of ∼E9.0 Sox1-Cre+/−;Z/EG+/− embryo. B Sagittal section of ∼E9.0 Bry-Cre+/−;Z/EG+/− embryo. Sections in ‘A’ and ‘B’ are oriented with the ventral side facing left. C Quantification of recombination frequency ∼E7.75 and ∼E9.0. D,E Localization of cells in which Bry-Cre induced recombination has occurred does not result in cellular diversion to neuroepithelium (Table S1U,V). D Sagittal sections of E9.5 embryos wherein one allele of Oct4 has been removed and the other is intact (Oct4Δ/+;Z/EG+/−;Bry-Cre+/−). GFP marks cells where recombination has occurred. E Sagittal section of E9.5 embryos wherein Oct4 has been depleted (Oct4Δ/f;Z/EG+/−;Bry-Cre+/−). The number of Bry+ cells are present in the neuroepithelium of E9.5 embryos is comparable to controls in ‘D’ which does not support diversion of cells into the neuroepithelium following Oct4 depletion. GFP marks cells where recombination has occurred. Magnified insets in the upper right correspond to the section outlined in each panel (D,E). Scale bars in ‘D’ and ‘E’ are 200 µm. (TIF) [file pgen.1003957.s007.tif]

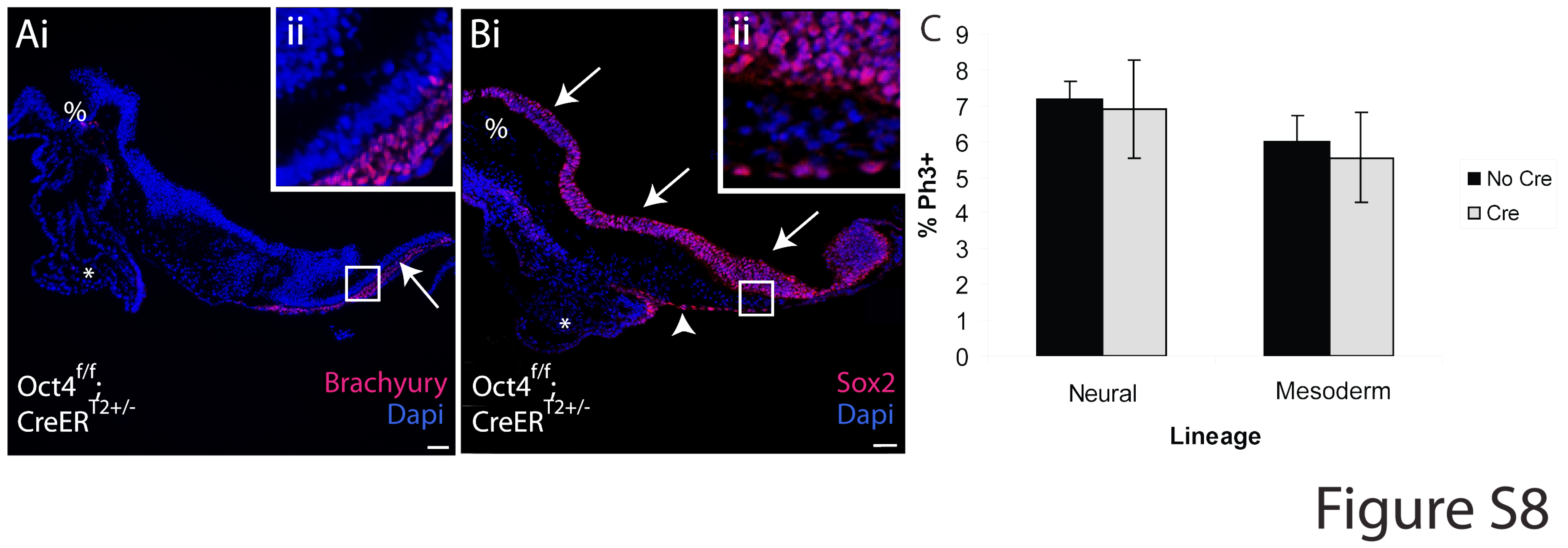

Supplement: Figure S8 — Specified lineages are present, appropriately localized, and proliferate at the same frequency as controls following Oct4 depletion (Table S1AC). The regions outlined with a white box in panel ‘i’ are magnified are magnified and provided as insets in panel ‘ii.’ Scale bars in ‘i’ are 50 µm, and are oriented such that the rostral end of the embryo is in the upper left of each panel ‘i’. The heart tube (*) and presumptive forebrain (%) are indicated to provide additional landmarks. A Brachyury is present in Oct4f/f;CreERT2+/− embryos 48 hrs ATA, visible here in trunk mesenchyme (arrow). B Sox2 is present throughout the neuroepithelium (arrows) and gut (arrowhead) in the posterior of Oct4f/f;CreERT2+/− embryos 48 hrs ATA. C Quantification of the fraction of lineage specified cells that are Ph3+ 48 hrs ATA (Oct4f/f;CreERT2+/− vs Oct4f/f). Data are presented as mean ±s.e.m. (TIF) [file pgen.1003957.s008.tif]

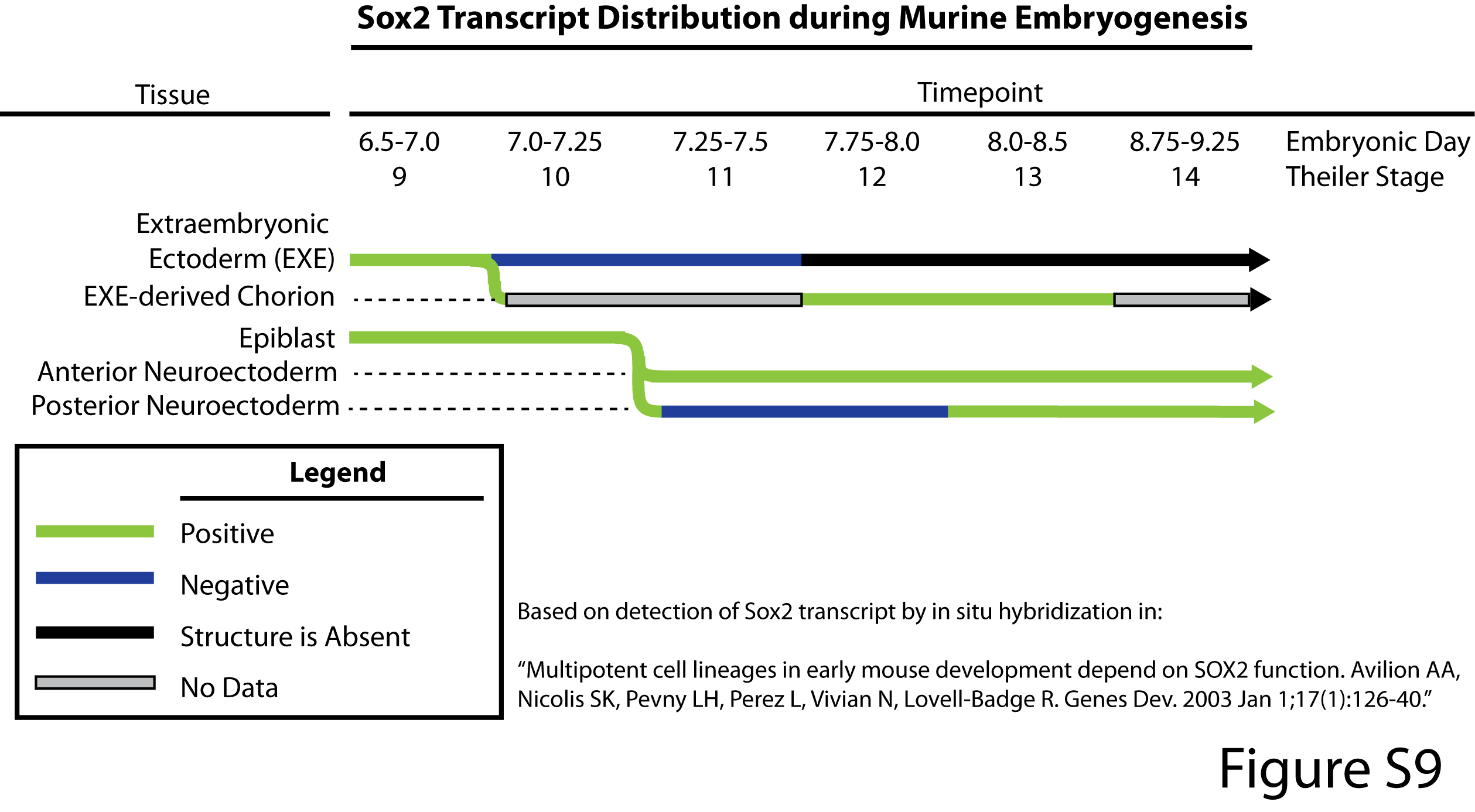

Supplement: Figure S9 — Sox2 transcript localization from E6.5–9.25 of murine development, based on [41]. (TIF) [file pgen.1003957.s009.tif]

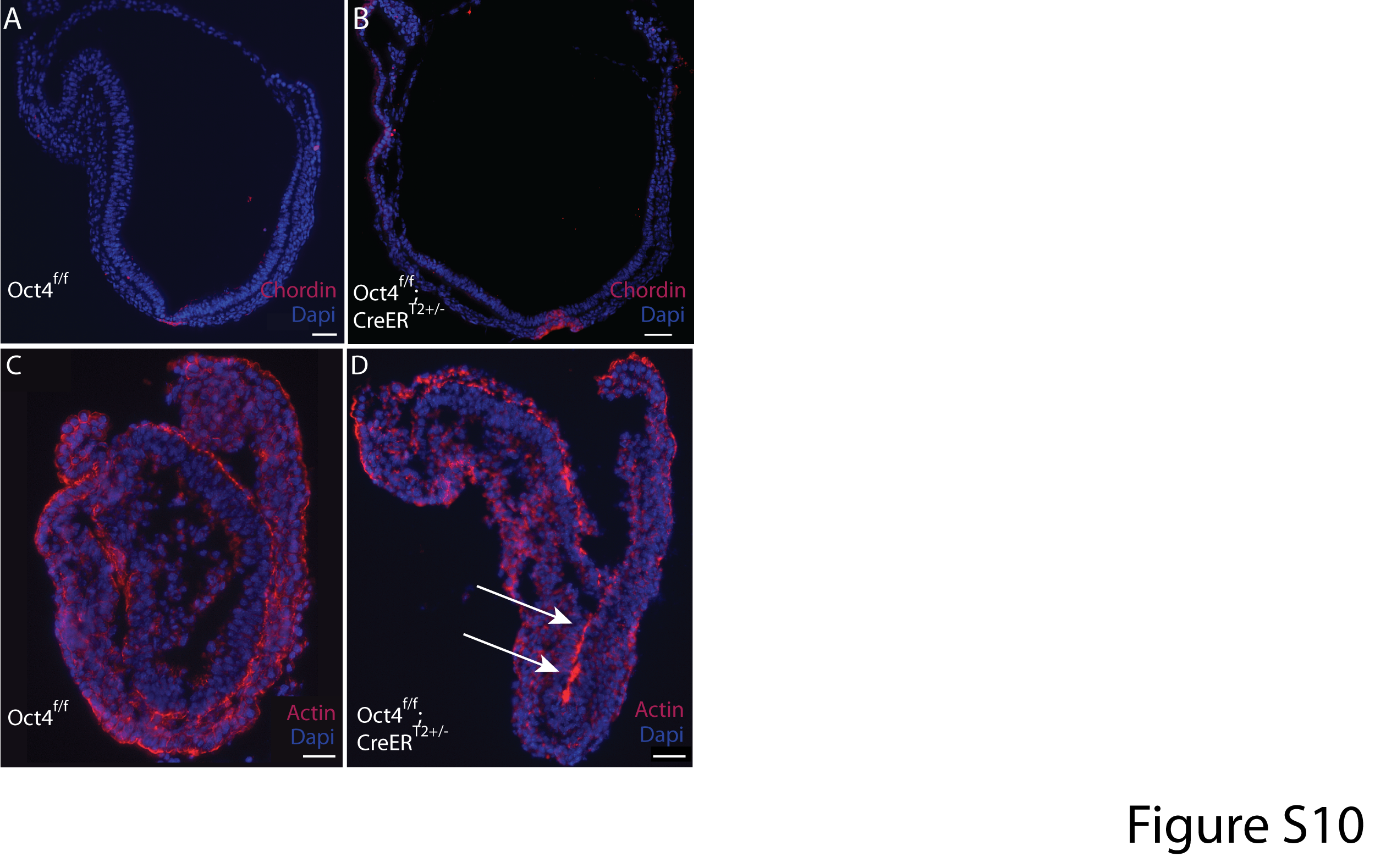

Supplement: Figure S10 — The Chordin domain persists, while actin filament distribution appears altered by immunohistochemistry in Oct4f/f;CreERT2+/− embryos following Oct4 depletion (Table S1AC). All litters depicted in panels ‘A–D’ were induced with tamoxifen ∼E7.0 and ∼E7.5. Scale bars in ‘A–D’ are 50 µm. A,B Specification of Chordin in the node occurs in spite of Oct4 depletion (36 hrs ATA). A Oct4f/f B Oct4f/f;CreERT2+/−. C,D The distribution of actin appears altered 24 hrs ATA in Oct4f/f;CreERT2+/− embryos. Arrows indicate region where anterior and posterior neuroepithelium of Oct4f/f;CreERT2+/− embryos may adhere. C Oct4f/f D Oct4f/f;CreERT2+/−. (TIF) [file pgen.1003957.s010.tif]

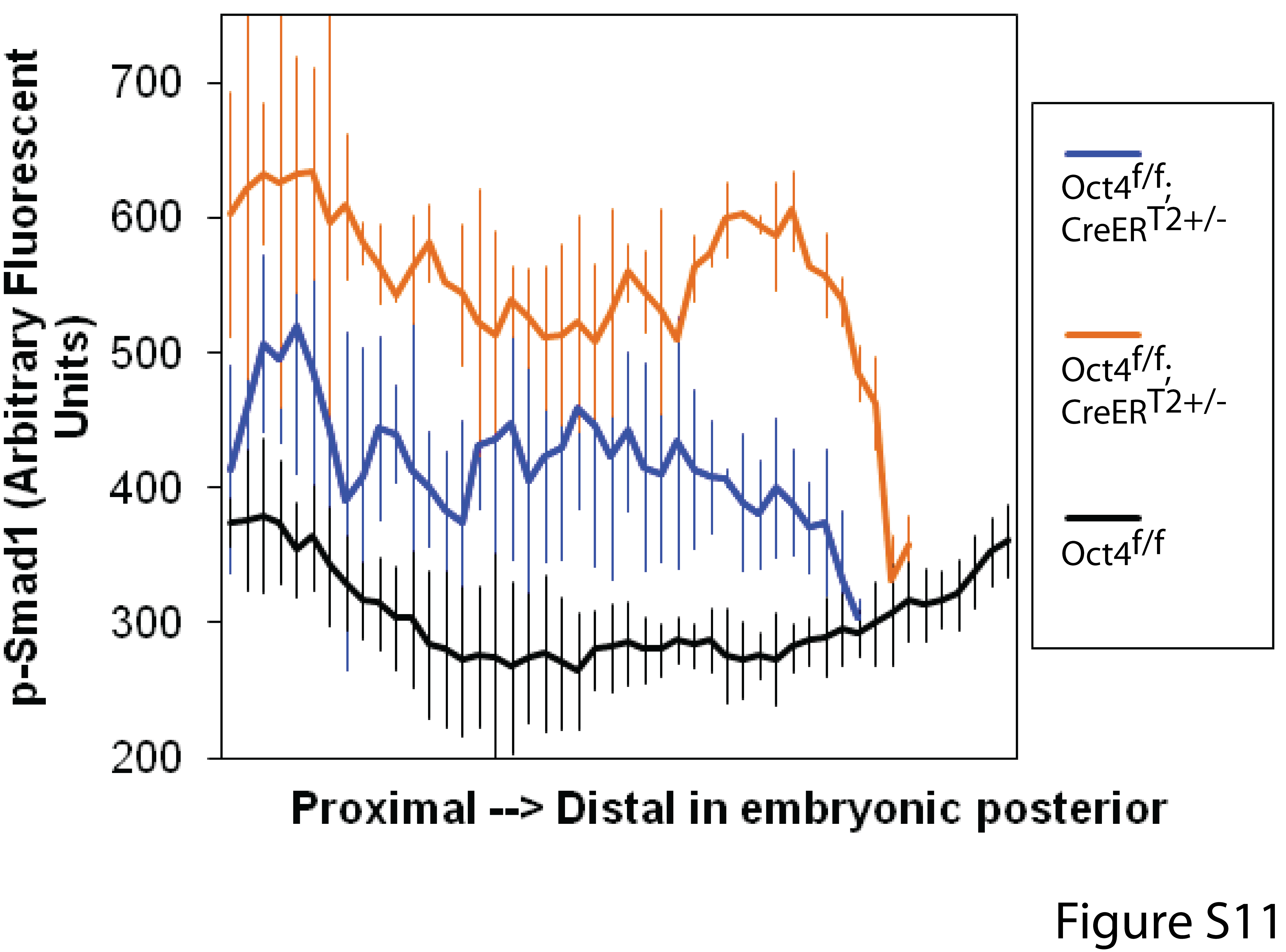

Supplement: Figure S11 — Quantification of p-Smad1 intensity in Oct4f/f; CreERT2+/− and Oct4f/f embryos 36 hrs ATA suggests increased p-Smad1 following Oct4 depletion. Mean p-Smad1 intensity (multiple sections of the same embryo) in embryonic posteriors is depicted. The plot is aligned such that the intensity of p-Smad1 plotted on the x-axis shows p-Smad1 intensity (left to right; proximal to distal) in the embryonic posterior. The y-intersect, estimated based on morphology, is roughly where the allantois ends and the embryonic posterior begins. (TIF) [file pgen.1003957.s011.tif]
