# Supplementary material for: Oct4 Is Required ∼E7.5 for Proliferation in the Primitive Streak
Source: PLoS Genet. 2013 Nov 14;9(11):e1003957. doi: 10.1371/journal.pgen.1003957 (PMC3828132; doi:10.1371/journal.pgen.1003957)
Supplement: Text S1 — Supplementary Methods. The supplementary methods section provides additional methodological detail related to: measurements made in the manuscript (timecourse of Oct4 protein depletion, mesenchyme density, neuroepithelial thickness, notochord thickness, distance between neural folds as well as Ph3+ and Caspase-3+ populations), Quantitative PCR (assay details), a summary of how chimeras were generated and the chimeric contribution quantified, detail concerning how the samples were prepared for microarrays and how the output of these assays was statistically analyzed. Supplementary references related to this methodology are also included. (DOC) [file pgen.1003957.s014.doc]

**Supplementary Methods**

*Oct4 is required ~E7.5 for proliferation in the primitive streak*

**Contents of Supplementary Methods**

**Basic Measurements…** page 1

**Oct4 depletion…** page 1

**Mesenchyme density…** page 2

**Neuroepithelium thickness…**  page 2

**Notochord thickness…** page 2

**Distance between neural folds…** page 2

**Marker+ populations (Ph3+ and Caspase-3+)…**  page 2

**Quantitative PCR…** page 3

**Generating Chimeras…** page 3

**Measuring percent chimerism in diploid chimeras…** page 4

**Microarray and Statistical Enrichment Analysis…** page 4

**Quantification of p-Smad1 Intensity** page 5

**Supplementary References** page 6

**Basic Measurements**

Oct4 depletion: The fraction of embryonic cells staining for Oct4 was counted manually. The data is represented as the fraction of nuclei (nuclei were assessed by visual inspection of either Dapi or Hematoxylin) that are Oct4+. Oct4+ and Oct4- cells were distinguished by visual inspection within each embryonic section.

Mesenchyme density: 50 x 50 μm outlines were made in 10 μm Hematoxylin and Eosin stained sagittal sections. The number of nuclei within a each was counted by eye and compared between Oct4^f/f^ and equivalent axially matched sections of Oct4^f/f^;CreER^T2+/-^ littermates.

Neuroepithelium thickness: 2 sets of repeated measures of neuroepithelial thickness were taken to obtain an average thickness for the distal neuroepithelium and thickness of adjacent neuroepithelium in each embryo. The average intra-embryo thickness (distal vs adjacent) was then compared.

Notochord thickness: The average number of nuclei in 10 μm Hematoxylin and Eosin stained transverse sections was counted by eye in axially matched sections and compared between Oct4^f/f^ and Oct4^f/f^;CreER^T2+/-^ embryos.

Distance between neural folds: Serial transverse sections of individual embryos were axially matched using the heart tube and section number. The average distance between neural folds at each axial level within each embryo was obtained and these averages used for the statistical comparison.

Marker+ populations (Ph3+ and Caspase-3+): Positive cells of each variety were scored by eye and the positive fraction calculated using the total population of cells (Dapi) or lineage specified fraction (eg Bry+) as appropriate.

**Quantitative PCR**

QPCR was performed using Taqman Assays (Applied Biosystems) on a 7900 HT Fast-Time PCR System (Applied Biosystems) and analyzed with software provided by the manufacturer: SDS 2.3. The following Taqman Assays were used: *Pou5f1* Mm00658129_gH, *Hprt* Mm00446968_m1, *Gusb* Mm01197698_m1, *Gusb* Mm01197698_m1, *Rplp0* Mm99999223_gH, *Sox2* Mm03053810_s1, *Smad1* Mm00484723_m1, *Cdx2* Mm00432449_m1*, Klf2* Mm01244979_g1, *Eomes* Mm01351984_m1, *Lefty2* Mm00774547_m1, *Dll1* Mm01279265_g1, *Bax* Mm01205547_g1, *Bcl2* Mm00477631 and *Cdkn1c* Mm01272135_g1.

**Generating Chimeras**

Chimeras were produced as outlined in [1]. In brief, females were superovulated with 5-10 IU Folligon (Intervet) and 5 IU Chorulon (Intervet) before flushing as 8-cell morula (E2.5) using M2 (Millipore). Zona pellucidas were removed using dose Tyrode’s Acidic Solution (Sigma) and morula aggregated overnight in KSOM +AA with D-Glucose (Millipore) under mineral oil (Millipore). All aggregates (~E3.5 blastocysts) were reimplanted into the uterus of E2.5 pseudopregnant females the following morning. Embryos were staged according to the uterine environment, such that reimplantation was considered E2.5. Contribution was assessed by semi-quantitative PCR. For tetraploid chimeras, 4-cell embryos were electrofused at 30V for 40usec, aggregated with RFP ES cells [2] once they reached the 8-cell stage, and then transferred into pseudopregnant females the following morning. To control for timing of development and tamoxifen administration in both the diploid and tetraploid experiments, embryos of different genotypes were mixed before transferring to surrogates and genotyped following dissection. All surrogate mothers were induced with tamoxifen at E6.0 and E6.5.

**Measuring percent chimerism in diploid chimeras**

Chimerism was assessed with the Oct4^f^ genotyping protocol [3]. It is a ‘competitive’ PCR reaction, eg the same primers yield an amplicon of different sizes for the endogenous and floxed *Pou5f1* allele. PCR on DNA extracted from heterozygous samples (Oct4^f/+^) yields both amplicons, however amplification of the more prevalent allele predominates when the allelic ratio of input DNA is unbalanced. We generated a standard curve by mixing DNA from CD1 (Oct4^+/+^) and Oct4^f/f^ mice: 100% CD1: 0% Oct4^f/f^, 90% CD1: 10% Oct4^f/f^….0% CD1:100% Oct4^f/f^, and assayed the range through which this genotyping protocol detects both alleles [3]. Below 20-60% Oct4 ^f/f^, e.g. 10% Oct4 ^f/f^, the floxed *Pou5f1*allele did not amplify and was not detected. Above this range, e.g. 80% Oct4 ^f/f^, the endogenous allele did not amplify and was not detected. Hence false negatives (eg low level chimerism) may have existed in the other 8 aggregation-derived embryos where the Oct4^f^ allele was not detected (Table S1AB).

**Microarray and Statistical Enrichment Analysis**

Embryos and extraembryonic tissue were dissected, marked as pairs and immediately flash frozen separately in liquid nitrogen. The extraembryonic tissues were used to infer the embryonic genotype. Embryos were then pooled according to their genotype, e.g. those with CreER^T2^ separated from those without for each timepoint. All samples at each timepoint had at least two embryos of either genotype. RNA was extracted with Trizol according to the manufacturer’s instructions (Invitrogen) and sent to the UHN Microarray Centre (Toronto, ON, Canada) for fluor-labeling (protocol GE2 v5.7), microarray hybridization, and array scanning. In brief, Cy3 and Cy5 (NEN Life Science) were incorporated into cDNA using Superscript II (Invitrogen) and fluor-labeled cDNA was purified using CyScribe GFX purification kit (GE). Hybridizations were conducted in DIG Easy Hyb solution (Roche) using fluor-reversed pairs (i.e. each sample was hybridized twice to two different arrays labeled once with Cy3 and once with Cy5) to Agilent 44k Mouse Gene 1.0ST Gene Chips, scanned using G2565C DNA Scanner, and features extracted using Agilent Feature Extraction Software as described in the UHN Microarray Centre’s version 10.5 (protocol GE2_105_Dec08). Intensities were averaged across fluor-flips prior to normalization. Data was normalized [4] and clustered using Rosetta Resolver. Enrichment for TF binding within manually identified clusters was assessed for previously defined genome binding coordinates [5] where a gene with transcription start site within 5 kB of a binding site was considered bound. Differential expression was defined using a p-value threshold of 0.01 (i.e. 1% of genes exceed this threshold in technical replicate hybridizations) and background comprised of genes denoted "present" by the software. For pathway enrichment, the following databases were used to define functional categories: GeneGo Pathways, Reactome, Kegg, NCI, Ingenuity Pathways, Biobase, and Biocarta.

**Quantification of p-Smad1 Intensity**

Fluorescence intensity of p-Smad1 was quantified using Cellomics ArrayScan™ V^TI^ platform (Thermo). In brief, the embryonic posterior of images captured with a Zeiss Axio Observer were cropped and oriented such that proximal to distal was left to right in Adobe Photoshop v12. Mean intensity values were binned amongst cells at equivalent positions on the proximal-distal axis within each section. Mean intensity values were averaged between sections of the same embryo to generate embryonic means, and plotted ± s.t.d.

**Supplementary References**

1. Wood SA, Allen ND, Rossant J, Auerbach A, Nagy A (1993) Non-injection methods for the production of embryonic stem cell-embryo chimaeras. Nature 365: 87-89.

2. Vintersten K, Monetti C, Gertsenstein M, Zhang P, Laszlo L, et al. (2004) Mouse in red: red fluorescent protein expression in mouse ES cells, embryos, and adult animals. Genesis 40: 241-246.

3. Kehler J, Tolkunova E, Koschorz B, Pesce M, Gentile L, et al. (2004) Oct4 is required for primordial germ cell survival. EMBO Rep 5: 1078-1083.

4. Weng L, Dai H, Zhan Y, He Y, Stepaniants SB, et al. (2006) Rosetta error model for gene expression analysis. Bioinformatics 22: 1111-1121.

5. Chen X, Xu H, Yuan P, Fang F, Huss M, et al. (2008) Integration of external signaling pathways with the core transcriptional network in embryonic stem cells. Cell 133: 1106-1117.
